# DDR1 regulates RUNX1-CBFβ to control breast stem cell differentiation

**DOI:** 10.1016/j.stemcr.2025.102576

**Published:** 2025-07-03

**Authors:** Colin Trepicchio, Gat Rauner, Nicole Traugh, Ruohong Wang, Meadow Parrish, Daniel E.C. Fein, Youssof Mal, Piyush B. Gupta, Stefano Monti, Charlotte Kuperwasser

**Affiliations:** 1 Department of Developmental, Molecular & Chemical Biology, Tufts University School of Medicine, Boston, MA 02111, USA; 2 Bioinformatics Program, Faculty of Computing & Data Science, Boston University, Boston, MA 02215, USA; 3 Laboratory for the Convergence of Biomedical, Physical, and Engineering Sciences, Tufts University School of Medicine, Boston, MA 02111, USA; 4 Section of Computational Biomedicine, Boston University Chobanian & Avedisian School of Medicine, Boston, MA 02118, USA; 5 Department of Biostatistics, Boston University School of Public Health, Boston, MA 02118, USA

**Keywords:** organogenesis, organoids, 3D model, organoid model, differentiation, adult stem cells, human breast, hydrogel model, tissue development, regeneration

## Abstract

Understanding epithelial stem cell differentiation and morphogenesis during breast tissue development is essential, as disruption in these processes underlie breast cancer formation. We used a next-generation single-cell-derived organoid model to investigate how individual stem cells give rise to complex tissue. We show that discoidin domain receptor 1 (DDR1) inhibition traps cells in a bipotent state, blocking alveolar morphogenesis and luminal cell expansion, which is necessary for complex epithelium formation. Disrupting RUNX1 function produced nearly identical phenotypes, underscoring its critical role downstream of DDR1. Mechanistically, DDR1 affects the interaction and expression of RUNX1 and its cofactor core binding factor beta (CBFβ), thereby regulating its activity. Mutational analyses in breast cancer patients reveal frequent alterations in the DDR1-RUNX1 signaling axis, particularly co-occurring mutations. Together, these findings uncover DDR1-RUNX1 as a central signaling pathway driving breast epithelial differentiation, whose dysregulation may contribute fundamentally to breast cancer pathogenesis.

## Highlights

• Primary breast organoids reveal facultative multilineage stem cells in adult tissues
• A novel DDR1-RUNX1 signaling axis regulates multiple stages of breast development
• A DDR1-RUNX1 axis regulates the stem-differentiation balance in breast organoid development

## Teaser

Kuperwasser and colleagues probed single-cell-derived miniature breast organoids to uncover a previously unknown link between DDR1 and RUNX1, in which activation of DDR1 drives RUNX1 to switch from stem cell to differentiation programming.

## Introduction

Epithelial organogenesis, morphogenesis, and differentiation are core processes of developmental biology, underlying the specification, expansion, and differentiation of stem cells into organized multicellular tissues. In the case of the human breast, which is characterized by both its complex ductal and lobular anatomy and its multilineage epithelium, the process by which these structures arise from stem cells and how they relate to each other during development has been difficult to study. In addition, how stem cell differentiation and tissue morphogenesis is controlled in a highly regenerative tissue such as the breast is important to understand as defects in these processes underlie the formation of breast cancer (Arendt et al., 2010, 2014; Russo and Russo, 2004).

The human breast is a dynamic tissue that undergoes several developmental phases, starting during embryogenesis with organogenesis, then continuing postnatally at puberty, again during pregnancy, lactation, and finally regressing during post-lactation involution. The breast actively regenerates during each menstrual and pregnancy cycle until ultimately atrophying and losing its regenerative capacity at menopause (Arendt et al., 2014; Russo and Russo, 2004). Unlike rodents, human breast tissue is composed of 15–20 lactiferous ducts, which are responsible for transporting milk from the lobules to the nipple. Lobules with differing levels of complexity are composed of many terminal ductal lobular units (TDLUs) that consist of terminal ducts culminating in grape-like clusters of alveoli (Arendt et al., 2014; Russo and Russo, 2004). A single lobule can have anywhere from 10 to 100 TDLUs, each composed of both luminal epithelial cells that line the inside of the ducts and alveoli and basal/myoepithelial cells that lie outside the luminal cells and are in direct contact with the basement membrane (Arendt et al., 2014; Russo and Russo, 2004; Woodward et al., 2005). During development, both luminal and myoepithelial cells are derived from bipotent stem cells that give rise to unipotent luminal and basal progenitors, which in turn serve as precursor reservoirs for mature luminal and myoepithelial cells in the adult tissue, respectively (Arendt et al., 2014; Woodward et al., 2005).

The ability of breast epithelial stem cells to create complex structural components, such as ducts and alveoli, is governed by a combination of intrinsic cell programming and cell-cell interactions, as well as extrinsic signals from the microenvironment (Woodward et al., 2005). Mouse models have been traditionally used to study mammary stem cells, development, and morphogenesis; however, there are inherent differences between species that limit the direct translation of findings to humans (Russo and Russo, 2004). Traditional models including 2D cell culture as well as 3D organoid and mammosphere models have also provided valuable insights in studying breast stem cells and epithelial differentiation. However, they fall short in capturing the complexities of human breast tissue organogenesis and morphogenesis.

Recently, we reported the creation of a next-generation 3D organotypic model with advanced representation of complex glandular human TDLU architecture and function (Rauner et al., 2021; Sokol et al., 2016). We found that human breast TDLU organoids form in response to collagen signaling through the activation of discoidin domain receptor 1 (DDR1) (Rauner et al., 2021). DDR1 activation by binding collagen gives rise to basal progenitors and luminal cells, which drive alveolar budding, branching, and the formation of complex TDLU organoids (Rauner et al., 2021). In this study, we investigated how DDR1 signaling in single stem cells leads to the creation of these complex multi-structural tissues. In doing so, we identified Runt-related transcription factor 1 (*RUNX1)* as a necessary transcriptional regulator linking DDR1 signaling with bipotent stem cell differentiation that in turn is required for tissue morphogenesis. These findings, along with mutational data, suggest that disruption of this signaling axis could have important implications in the biology of breast cancer.

## Results

### Live imaging of single cells reveals the complex and dynamic nature of TDLU organoid formation and morphogenesis

**Figure 1:**
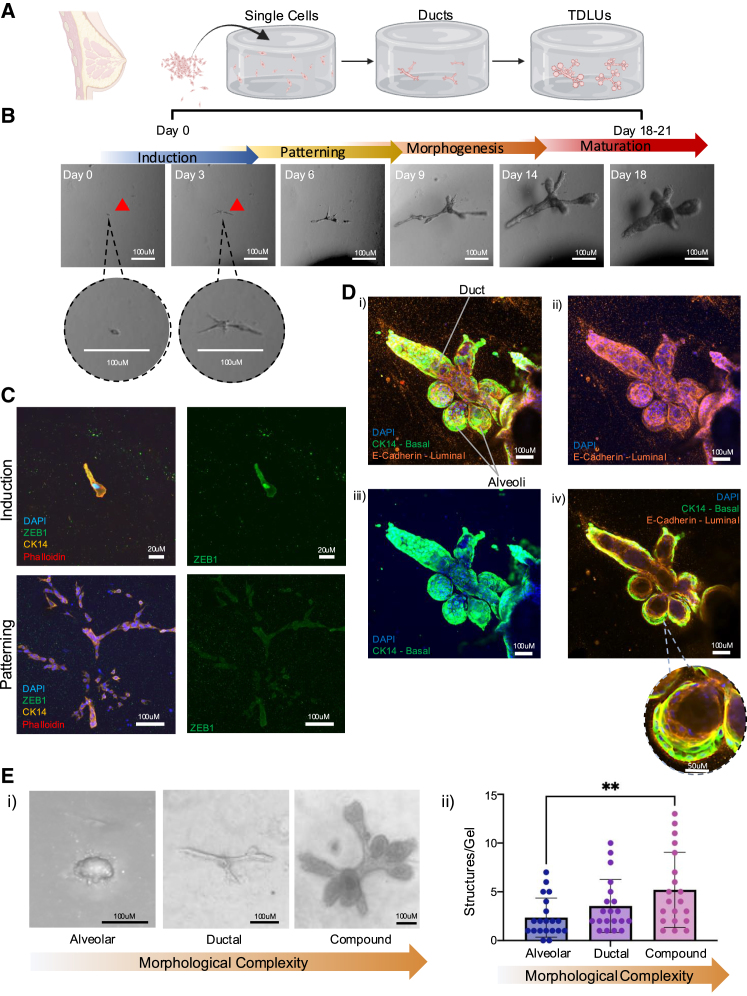
Phenotypic characterization of patient-derived single-cell hydrogel methodology (A) Schematic representation and summary of human breast organogenesis in a 3D hydrogel TDLU organoid model. (B) Timeline with brightfield images of TDLU formation from single cells, starting on day 0 immediately after hydrogel polymerization to the formation of complex ductal-lobular structures from day 18 onward. Four phases of organogenesis showing induction, patterning, morphogenesis, and maturation. Scale bars, 100 μm. (C) Representative immunofluorescent staining showing single cells expressing EMT transcription factor ZEB1 during induction, while during patterning this expression is lost. ZEB1 (green), CK14 (orange), phalloidin (red), and DAPI (blue). Scale bars, 20 μm for induction and 100 μm for patterning. (D) Immunostaining of (i–iv). A three-dimensional maximum intensity projection (MIP) TDLU organoids, at day 21 of development, stained with phalloidin (red) for actin cytoskeleton, with CK14 (green), E-Cadherin (orange) for cell lineage, and with DAPI (blue) for nuclei. Scale bars, 100 μm. (v) A two-dimensional cross-section of MIP (i) with offset highlighting a lobule showing distinct CK14^+^ (green) and E-Cad^+^ (orange) layers with an interacting layer displaying yellow. Scale bars, 100 μm; scale bars of offset, 50 μm. (E) Representative brightfield images (i) and quantification (ii) of 3D organoid morphologies from 5 primary patients. Mean ± SD (*n* = 4 gels/primary patient samples). Scale bars, 100 μm.

To investigate stem cell dynamics and tissue morphogenesis from the earliest stages of breast development, we seeded single primary human breast epithelial cells from various patients (*N* = 7; see Table S1) into 3D hydrogels. We observed the formation of complex, multilayered, and heterogeneous breast TDLU organoids in all donor samples (Figure 1A) (Rauner et al., 2021; Sokol et al., 2015, 2016). We combined this method with high-speed point scanning confocal microscopy, to visualize organogenesis from a single bipotent stem cell in real time (Figure 1B; Video S1). We observed that TDLU organoid formation takes ∼18–21 days to complete and proceeds through 4 distinct stages: induction, patterning, morphogenesis, and maturation. Within the first 3 days after seeding, stationary single stem cells are induced to proliferate and exit the bipotent stage (Figure 1B; Video S1) (Rauner et al., 2023). Shortly thereafter, between days 5 and 8, stem cell progeny exhibit dynamic cell movements where they travel along an emerging branch prior to the formation of a cohesive tissue (Figure 1B; Video S1) (Rauner et al., 2023). Interestingly, while single cells during the induction phase express the epithelial-mesenchymal transition (EMT)/stem cell marker ZEB1, by the patterning phase, ZEB1 expression is lost, consistent with stem cells undergoing lineage commitment and differentiation (Figure 1C). Epithelial cells continue to divide and invade the matrix, in a manner reminiscent of tissue patterning, clearing room for the organoid to develop primary ducts. Around day 9, basal progenitor cells begin the process of morphogenesis whereby ducts elongate and alveolar buds appear (Figure 1B; Video S1) (Rauner et al., 2023). This leads to the formation, maturation, and differentiation of organoids that are anatomically equivalent to human breast TDLUs (Figures 1Di–1Div). These structures are histologically normal with an inner layer containing mature E-Cadherin expressing luminal cells (Figures 1Di, 1Dii, and 1Div) and external CK14^+^ myoepithelial cells (Figures 1Di, 1Diii, and 1Div). Since TDLU organoids are derived from a single cell (Video S1) (Rauner et al., 2023), and the resulting complex tissue is composed of both ducts and alveoli as well as both basal and luminal cell lineages, (Figures 1B and 1D), this parent-progeny relationship between the precursor cell and the TDLU demonstrates they are facultative multilineage stem cells (Rauner et al., 2023; Woodward et al., 2005).

Video S1. Confocal Imaging Time-Lapse of a Single Human Breast Cell Generating a Mature Ductal-Lobular Organoid

Interestingly, not all organoids formed from single cells result in highly complex TDLUs. Rather, some remain in a simpler state, categorized as alveoli only or ductal only, which is not observed when seeding tissue clusters (Rauner et al., 2021) (Figure 1E). Alveolar organoids are characterized by the formation of simple acini or clusters of cells, while ductal organoids are characterized by the formation of elongated and branched structures and lack alveoli. In contrast, complex TDLU organoids are compounded structures containing both ducts and varying numbers of alveoli at their terminal regions (Figure 1E). A quantitative assessment of organoid types across five patient samples revealed that single cells form on average 11 organoids per 1,000 seeded cells, with a distribution of 2.5 alveolar, 3.5 ductal, and 5.5 compound TDLU structures, respectively (Figure 1E).

### Inhibition of DDR1 restricts stem cells to a bipotent state and blocks alveologenesis

**Figure 2:**
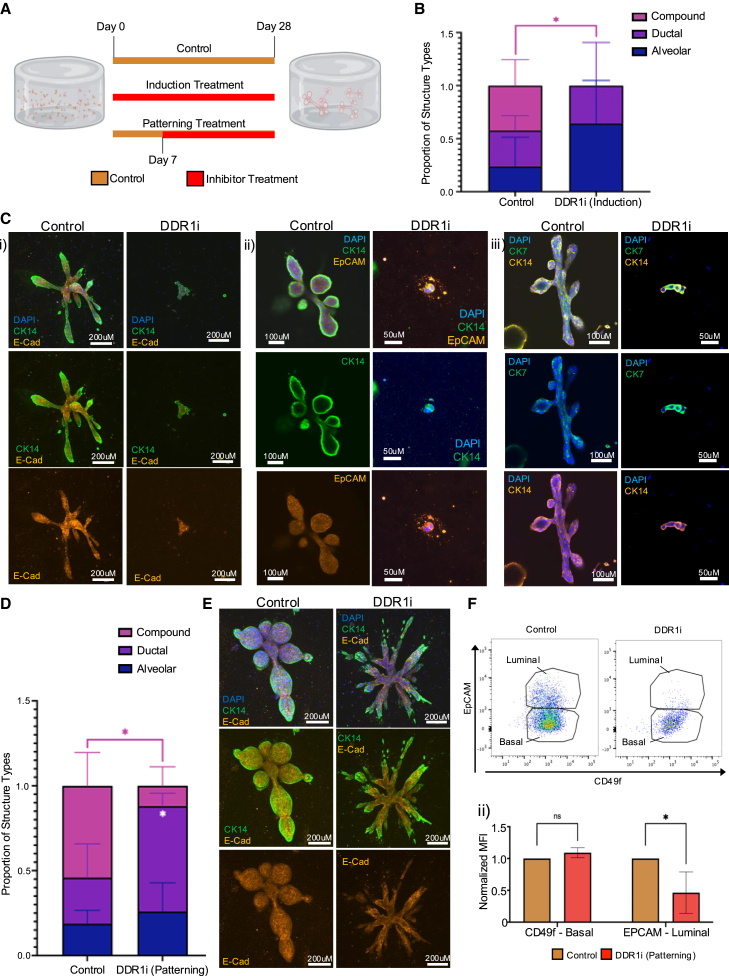
Effect of DDR1 signaling inhibition on TDLU organoid development (A) Schematic of the strategy to test the effects of DDR1i on breast TDLU organogenesis. Inhibition during induction began with DDR1i treatment starting at day 0 and concluded when control structures were fully formed no later than day 28. Inhibition during patterning started with DDR1i beginning day 7 and concluding around day 28. (B) Normalized quantification of the types of organoids that formed following DDR1i treatment during induction. Data are presented as mean ± SD (*n* = 4 gels/3 primary patient samples). (C) Representative immunofluorescence staining of organoids from control or DDR1 inhibitor-treated gels, with treatment initiated during the induction phase of organoid formation. Stained as follows: (i) basal marker CK14 (green), epithelial marker E-Cadherin (orange), and DAPI (blue) staining (scale bars, 200 μm); (ii) basal marker CK14 (green), luminal marker EpCAM (orange), and DAPI (blue) staining (scale bars, 100 and 50 μm for control and DDR1i treatment, respectively); (iii) luminal marker CK7 (green), basal marker CK14 (orange), and DAPI (blue) staining (scale bars, 100 and 50 μm for control and DDR1i treatment, respectively). (D) Normalized quantification of the types of organoids formed in DDR1i treated cultures beginning during patterning. Data are presented as mean ± SD (*n* = 4 gels/3 primary patient samples). (E) Representative immunofluorescence staining of organoids from control or DDR1i treated gels initiated during the patterning phase of organoid formation. CK14 (green), E-Cadherin (orange), and DAPI (blue) staining. Scale bars, 200 μm. (F) Representative flow cytometry plot analysis of basal (CD49f) and luminal (EpCAM) cells (FACS) (i) and quantification of mean fluorescent intensity (ii) from primary patient samples cultured in 3D, treated with DDR1i during patterning (*n* = 3 primary samples, and values are expressed as mean ± SD). Statistical significance was determined via multiple t tests, with significance levels indicated as follows: ^∗^*p* < 0.05, ^∗∗^*p* < 0.01, ^∗∗∗^*p* < 0.001, and ^∗∗∗∗^*p* < 0.0001.

Using this approach, we examined the effects of DDR1, a receptor tyrosine kinase activated by collagen binding, on the early stages of development, using the well-studied specific small molecule inhibitor of DDR1, DDR1-IN-1 (DDR1i) (Rauner et al., 2021) (Figure 2A). Prior studies have established a role for DDR1 during mammary morphogenesis (Rauner et al., 2021) but little is known of its role in stem cells during the early stages of breast development. When single patient-derived cells were treated with DDR1i during induction, there was a complete absence of TDLU formation, a large increase in the proportion of alveolar only organoids in relation to other structure types, and a significant reduction in the overall number of organoids formed (Figures 2B and S1A). Additionally, alveolar-only organoids that did form in the presence of DDR1i at induction exhibited noticeable abnormalities. Staining of these disorganized, rudimentary structures with epithelial marker E-Cadherin along with specific luminal (EpCAM and CK7) and basal (CK14) markers (Figure 2C) revealed that cells within the structures co-stained for both luminal and basal markers, suggesting that they are trapped in a bipotent state. Interestingly, these simple acinar structures no longer express nuclear ZEB1 (Figures S1Bi and S1Bii).

To assess the role of DDR1 during the patterning stage, 3D cultures derived from single cells were treated with DDR1i starting on day 7 (Figure 2A). Although the total number of organoids formed was comparable to the control, these organoids also failed to develop into complex ductal-alveolar TDLUs (Figures 2D and S1C). Instead, these organoids remained in a simple ductal-only in state, consistent with DDR1i treatment on tissue clusters (Rauner et al., 2021). Staining of these ductal-only organoids with E-Cadherin and CK14 demonstrated lineage specification, and differentiation into both ductal luminal and basal cells (Figure 2E). This indicates that DDR1 inhibition during patterning does not alter stem cell differentiation but does interfere with tissue patterning and the formation of alveoli.

The capacity to undergo alveolar morphogenesis is driven by coordinated expansion and differentiation of luminal cells (Rauner et al., 2021). In their absence, ductal structures form. Therefore, we examined whether the lack of alveolar morphogenesis in response to DDR1i might be due to a failure of luminal cell expansion. Luminal (EpCAM^high^) and basal (EpCAM^neg/low^/CD49f^pos^) cells were assessed by flow cytometry in primary single-cell-derived organoids that formed in the presence or absence of DDR1i during patterning. Indeed, a significant reduction in the number of luminal cells was found in DDR1i-treated organoids (Figure 2F), consistent with the previous data indicating that the failure to form alveoli is due to a failure in luminal cell expansion (Rauner et al., 2021). Together, these findings demonstrate that DDR1 plays a crucial role in driving the specification and differentiation of stem cells during induction but also the expansion of luminal cells during patterning.

### DDR1 regulates *RUNX1* expression and transcriptional activity

**Figure 3:**
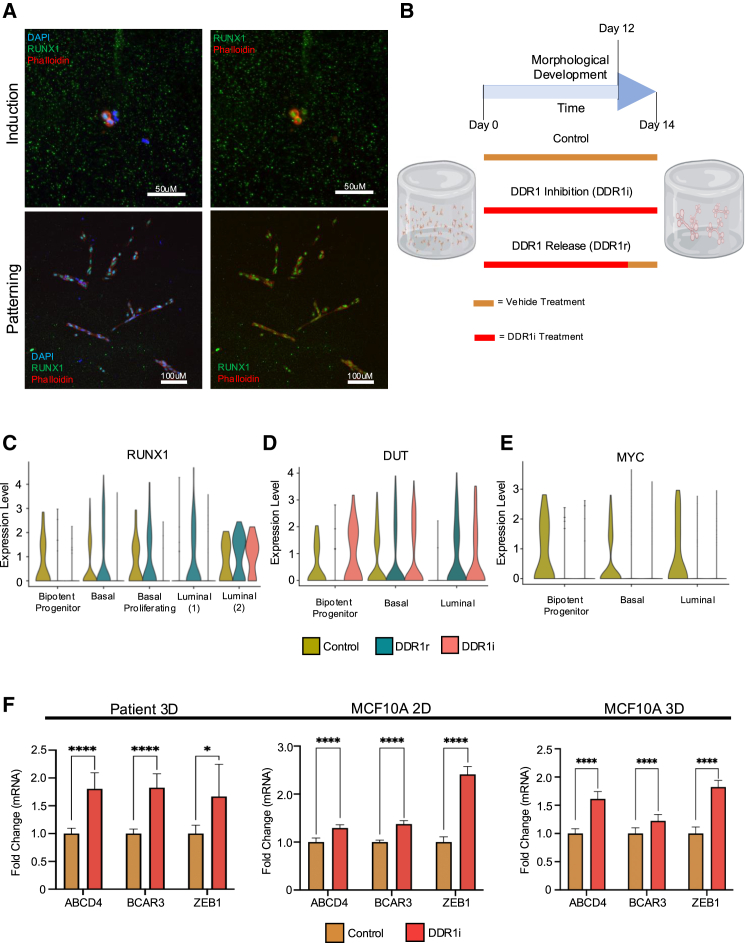
Effect of DDR1 inhibition on *RUNX1* and RUNX1 target genes (A) Representative immunofluorescent panels showing RUNX1 staining in developing organoids. Scale bars, 50 and 100 μm for induction and patterning time points, respectively. (B) Schematic representation of DDR1 inhibitor time course design for scRNA-seq. DDR1i treatment starting on day 0 and concluding on day 14, and DDR1r treatment with DDR1i initiation on day 0 and cessation on day 12, with the last two days free from inhibition. (C–E) Violin plots showing the distribution of *RUNX1*, *DUT*, and *MYC* expression in primary tissue organoids under control, DDR1r, or DDR1i conditions. (F) Compilation of RT-qPCR quantification data derived from three primary patient samples cultured in a 3D environment, MCF10A cells grown in 2D or MCF10A cells grown in 3D, comparing RUNX1 target gene response to DDR1 inhibition. Values are expressed as mean ± SD. Statistical significance was determined via multiple t tests, with significance levels indicated as follows: ^∗^*p* < 0.05, ^∗∗^*p* < 0.01, ^∗∗∗^*p* < 0.001, and ^∗∗∗∗^*p* < 0.0001.

Studies have suggested that *RUNX1* is essential for breast stem cells to exit the bipotent state and commit to a specific lineage (Hong et al., 2017; Sokol et al., 2015). Consistent with these findings, Runx1 protein expression was observed during late induction and it remained elevated throughout the subsequent stages of organogenesis (Figures 3A and S2). Since DDR1i inhibited lineage specification and differentiation in stem and progenitor cells, we therefore examined *RUNX1* expression using single-cell RNA sequencing (scRNA-seq). Organoid cultures were grown for 14 days and treated continuously with DDR1i (DDR1i) or treated with DDR1i for 12 days followed by release from inhibition for two additional days (DDR1r) before sequencing (Figure 3B) (Rauner et al., 2021).

Cells were categorized into clusters based on their expression profiles as bipotent progenitors, non-dividing basal cells (basal), proliferating basal cells, and two types of luminal cells (luminal 1 and luminal 2). Luminal 1 cells were characterized by expression of classical luminal epithelial markers such as *KRT7*, *KRT8*/18, *KRT19*, and *EPCAM* (Rauner et al., 2021; Sokol et al., 2016), while luminal 2 cells, expressed more mature luminal differentiation markers including Lactotransferrin (Figure S3A; Table S2).

Consistent with the immunofluorescence (IF) findings, in the absence of DDR1i, *RUNX1* is heterogeneously expressed in various cell types including bipotent stem cells, basal cells, and luminal cells (Figure 3C). The highest levels of *RUNX1* are expressed in bipotent progenitor cells and basal cell types, with lower levels expressed in luminal cells. Interestingly, DDR1 is expressed in similar cell populations including bipotent progenitor and basal cells with lower levels in luminal cells (Figure S3B).

Notably, treatment with DDR1i resulted in a significant reduction of *RUNX1* expression in both basal cell populations and bipotent progenitor cells. This downregulation was reversible in basal cells, as *RUNX1* expression recovered upon removal of DDR1i (DDR1r) (Figure 3C). However, bipotent progenitors exhibited an irreversible decrease in *RUNX1* expression, suggesting distinct regulatory mechanisms influenced by DDR1 in these cell types. Interestingly, luminal 2 cells, which uniquely do not express DDR1 in the breast, were also the only cell type unaffected by DDR1i in terms of *RUNX1* expression (Figures 3C and S3B). This observation underscores the differential roles of DDR1 across various breast cell lineages.

Given that *RUNX1* expression levels were sensitive to DDR1 activity, we also examined whether *RUNX1* target gene expression might be responsive to DDR1i. Indeed, DDR1i caused differential expression in several direct *RUNX1* target genes including *DUT*, an essential nucleotide metabolism enzyme seen upregulated across breast cancers (Davison et al., 2021), and *MYC*, along with *ANP32B*, *PLEC*, *CEBPD*, *ID1*, and *STAT3* (Figures 3D, 3E, and S3C–S3G). The responsiveness of *RUNX1* to DDR1i was specific as expression of other members of the RUNX family (*RUNX2* and *RUNX3*) (Figures S3H and S3I) or its essential cofactor core binding factor beta (*CBFβ*) (Figure S3J) were not affected by DDR1i.

To further validate these findings, we treated three additional primary organoid cultures and assessed mRNA expression of additional *RUNX1* target genes to DDR1i. While patient heterogeneity in response to DDR1i was observed (Figures S3Ki–S3Kiii), *RUNX1* target genes were similarly affected by DDR1i (Figure 3F). We also examined the expression of these *RUNX1* target genes in MCF10A and MCF10F cells, normal immortalized breast cell lines that maintain populations of basal and luminal lineages (Sokol et al., 2015). Cultured in 2D and stimulated with collagen and DDR1i, MCF10A (Figure 3F) and MCF10F (Figure S3L) cells showed a significant change in mRNA expression of these *RUNX1* target genes. MCF10A cells grown in 3D also showed a significant change in *RUNX1* target gene expression (Figure 3F). Taken together, these data show that DDR1 activity can modulate *RUNX1* expression and the expression of *RUNX1* target genes.

### Disruption of RUNX signaling phenocopies DDR1 inhibition

**Figure 4:**
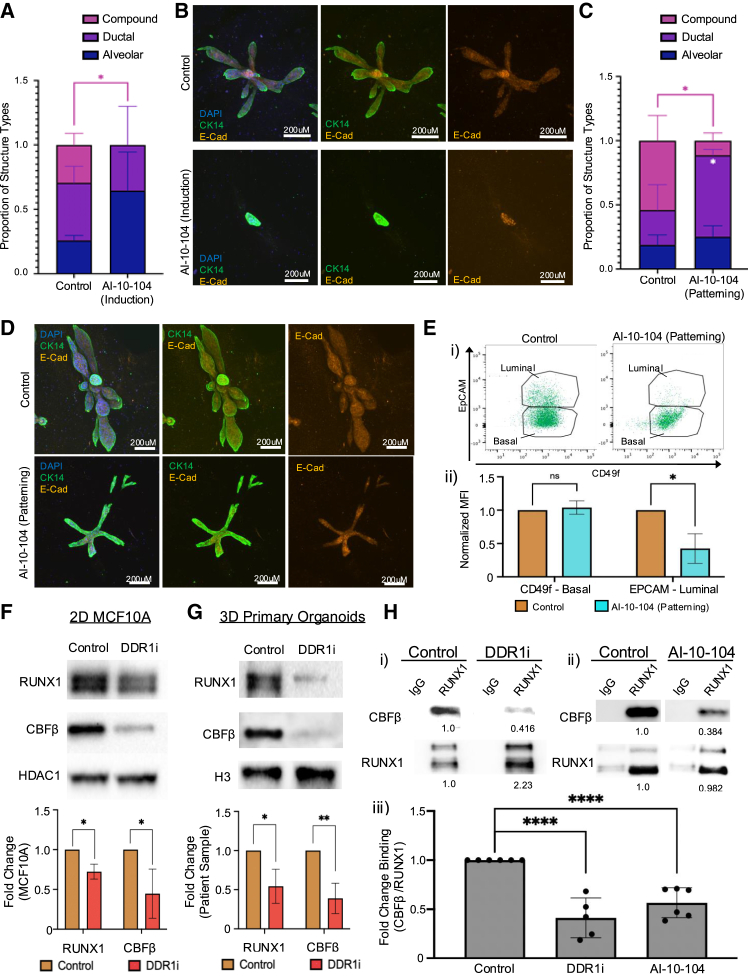
Effect of RUNX signaling inhibition on TDLU organoid development (A) Quantification of the types of organoids that formed following AI-10-104 treatment during induction. Data are presented as mean ± SD (*n* = 4 gels/2 primary patient samples). (B) Representative immunofluorescence staining of organoids from control or RUNX inhibitor-treated gels, with treatment initiated during the induction phase of organoid formation. CK14 (green), E-Cadherin (orange), and DAPI (blue) staining. Scale bars, 200 μm. (C) Quantification of the types of organoids that formed following AI-10-104 treatment during patterning (*n* = 4 gels/3 primary patient samples). Data are presented as mean ± SD. (D) Representative immunofluorescence staining of organoids from control or RUNX inhibitor-treated gels, with treatment initiated during the induction phase of organoid formation. CK14 (green), E-Cadherin (orange), and DAPI (blue) staining. Scale bars, 200 μm. (E) Representative flow cytometry plot analysis of basal (CD49f) and luminal (EpCAM) cells (FACS) (i) and quantification of mean fluorescent intensity (ii) from primary patient samples cultured in 3D, treated with RUNX inhibitor during patterning (*n* = 3 primary samples). values are expressed as mean ± SD. (F) Representative western blot analysis and quantification of Runx1 and Ddr1 expression from nuclear lysates of MCF10A cells treated with collagen and DDR1i (*n* = 3). Data are presented as mean ± SD. (G) Representative western blot and quantification of nuclear protein from fractionated lysate obtained from primary patient samples cultured in 3D hydrogels (*n* = 3). Data are expressed as mean ± SD. (H) CoIP of Runx1 and subsequent blotting and quantification of CBFβ from the lysate of MCF10A samples cultured in 2D treated with collagen or collagen and either DDR1i or RUNX inhibitor AI-10-104. Normalized quantification values below the blot. Quantification is derived from *n* = 6 independent experiments for control and AI-10-104 and *n* = 5 for DDR1i treatment and is presented as mean ± SD. Statistical significance was determined through multiple t tests, with significance levels indicated as follows: ^∗^*p* < 0.05, ^∗∗^*p* < 0.01, ^∗∗∗^*p* < 0.001, and ^∗∗∗∗^*p* < 0.0001.

Given that DDR1 regulates the expression of *RUNX1* and its target genes, and considering RUNX1’s role in breast stem cell differentiation (Sokol et al., 2015), we investigated whether inhibiting RUNX1 could similarly impact organoid formation, as observed with DDR1i. To this end, we treated primary single-cell-derived organoid cultures with the pan-RUNX inhibitor AI-10-104, which impedes RUNX activity by blocking its binding to CBFβ (Figure S4A) (Illendula et al., 2016). Although AI-10-104 is a broad-spectrum RUNX inhibitor, RUNX1 is the only RUNX family member expressed in these organoids, thus likely enhancing the specificity of the observed effects to RUNX1 (Figures S3H and S3I). AI-10-104 treatment was initiated either on day 0, before stem cells exit from bipotency, or on day 7 during progeny patterning. Similar to DDR1i, administering AI-10-104 during induction led to an increase in the proportion of alveolar-only organoids compared to other structures, as well as a complete loss of formation of mature TDLU organoids (Figure 4A). However, unlike DDR1i, there was no significant reduction in the total number of organoids that formed (Figure S4B). The alveolar colonies that did develop in the presence of AI-10-104 were immature, disorganized, and composed of cells that are double-positive for both luminal and basal markers, consistent with stem cells being trapped in a bipotent state (Figure 4B) (Sokol et al., 2015).

Similar to DDR1i, AI-10-104 treatment during the patterning phase also led to a loss of complex TDLU organoid formation (Figure S4C). The organoids that did form under AI-10-104 treatment were primarily composed of simple ductal structures (Figure 4C). Despite these changes, the total number of organoids remained unaffected by AI-10-104 treatment (Figure S4D), suggesting a blockage in tissue patterning rather than in organoid initiation. Immunofluorescence staining for E-Cadherin and CK14 in organoids treated with AI-10-104 starting from day 7 revealed lineage specification similar to that observed with DDR1i treatment, despite the inability to form alveoli at the terminal ends of the elongating ductal structures (Figure 4D). We also examined whether the lack of alveolar morphogenesis in AI-10-104 treated organoids might be due to differences in the number of cells present within the structures. Indeed, a significant reduction in the number of EpCAM^high^ luminal cells was found in AI-10-104 treated organoids (Figure 4E), consistent with the notion that the failure to form alveoli is due to a failure of luminal cell expansion (Rauner et al., 2021).

Since DDR1i and AI-10-104 treatment phenocopy each other, we sought to determine whether DDR1 inhibition might affect the expression and/or interaction of RUNX1 and CBFβ. MCF10A cells stimulated with collagen and DDR1i showed a significant reduction in the total levels of RUNX1 protein expression as well as the expression of RUNX1 co-factor CBFβ (Figure 4F), whose binding is essential for both RUNX1 protein stabilization and transcriptional activity (Qin et al., 2015). Similarly, primary single-cell-derived organoids grown in 3D treated with DDR1 inhibitor also exhibited a similar decrease of RUNX1 and CBFβ protein expression (Figure 4G).

The RUNX1-CBFβ complex is critical for both the stability of RUNX1 protein but also for its activity as a transcription factor (TF) (Qin et al., 2015). Since DDR1i reduced the levels of RUNX1 and CBFβ, we investigated whether DDR1 inhibition might also affect their interaction. Indeed, oc-immunoprecipitation of CBFβ with RUNX1 in MCF10A cells showed a significant reduction in complex formation in the presence of DDR1i (Figures 4Hi and 4Hiii), similar to that seen with the RUNX1-CBFβ inhibitor AI-10-104 (Figures 4Hii and 4Hiii; Figure S4A).

We used two additional RUNX inhibitors, with differing effects on RUNX1/CBFβ complex formation to assess morphological phenotypes. Cells and organoids were treated with either RO5-3335, which was discovered in a screen for small molecules that also disrupts the RUNX1 and CBFβ complex (Figure S4A) (Illendula et al., 2016), or AI-10-49, which targets the oncogenic CBFβ fusion protein CBFβ-SMMHC more selectively than the wild-type complex. RO5-3335 treatment phenocopied DDR1i or AI-10-104 treatment and had a significant increase in the proportion of alveolar or ductal structures generated when treatment began during induction or patterning, respectively, along with a loss of complex TDLU structures (Figures 2B, 4A, S4A, and S4E). While treatment with AI-10-49 during induction also resulted in the loss of complex TDLU structures, it caused significantly different structural proportions in culture compared to other treatments. The notably similar phenotypes of organoids treated between DDR1i, where DDR1i decreases CBFβ/RUNX1 expression, and inhibitors specific for CBFβ/RUNX1 complex formation (AI-10-104 and RO5-3335), suggest that DDR1 regulates RUNX expression and activity to influence tissue patterning and differentiation.

Finally, we also treated organoids with a gamma-secretase inhibitor to block NOTCH1 signaling during the same experimental time points to rule out confounding effects from overlapping stem cell signaling pathways and evaluate whether all inhibitors of stem cell activity phenocopy each other. The Notch signaling pathway plays a crucial role in the regulation of mammary stem cells and promotes the differentiation of luminal progenitors while inhibiting the myoepithelial lineage, thereby helping to shape the architecture of the mammary gland (Rauner et al., 2021). Interestingly, inhibiting NOTCH1 during induction led to a failure in TDLU formation but did not cause an expansion of alveolar organoid formation, an outcome not observed with either DDR1i or AI-10-104 treatments (Figure S4F). Instead, NOTCH1 inhibition during both induction and patterning led to an increase in ductal-only organoids compared to other structure types. This indicates that stem cell activity regulated by NOTCH during the early stages of breast organoid development differs from that of DDR1-RUNX1, highlighting the distinct stem cell signaling pathways influencing tissue development and architecture.

Together, these results elucidate a common stem cell regulatory pathway involving these two proteins, which differs from Notch signaling that guide the complex morphological development of stem cells into complex breast tissue organoids. It also underscores the importance of RUNX during induction and patterning. Like DDR1, during induction, RUNX1 is necessary for differentiation of bipotent stem cells, while during patterning, it is required for the expansion of luminal cells. The disruption of this luminal cell differentiation consequently inhibits alveologenesis, preventing the formation of complex TDLUs and thus mirroring the effects observed with DDR1 inhibition.

### DDR1 and RUNX share a core transcriptional network that controls breast epithelial differentiation

**Figure 5:**
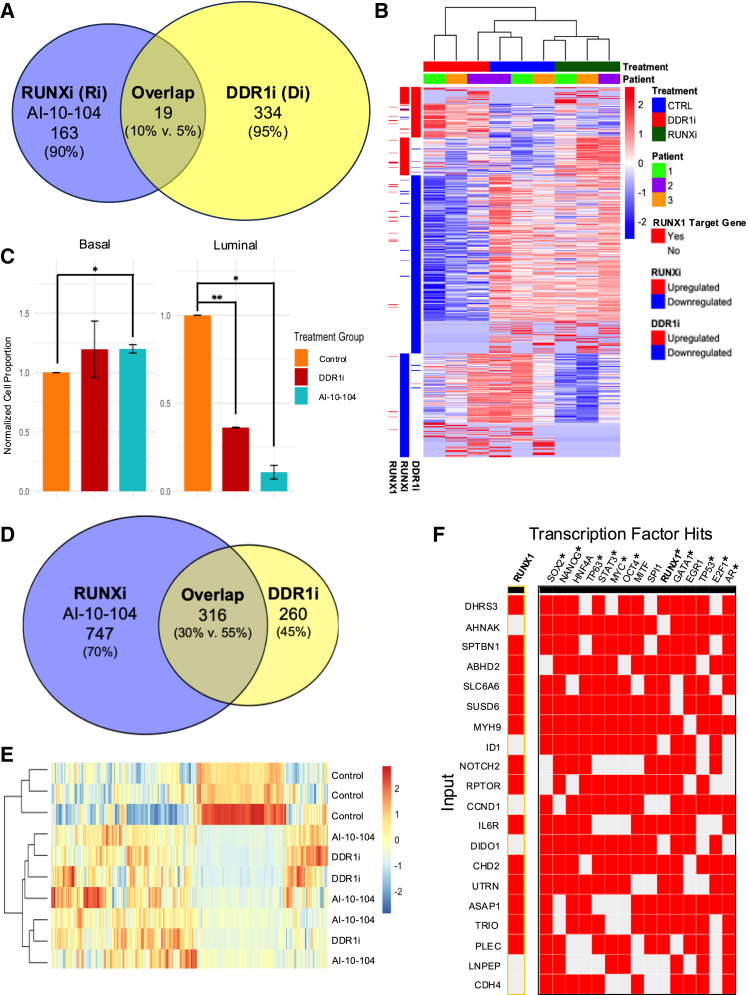
Interplay between DDR1 and RUNX1 in regulating breast epithelial differentiation (A) Venn diagram illustrating the overlap of differentially expressed genes in three primary patient treated with DDR1 and RUNX inhibitors. (B) Heatmap of the overlapping differentially expressed genes from DDR1i and RUNXi treated organoids, as compared to the control. Bars on right represent clustered groupings of differentially expressed genes for DDR1i and RUNXi. Dashes in *RUNX1* column represent *RUNX1* target genes. (C) Normalized cell populations determined through the deconvolution of bulk sequencing by using CIBERSORTx (Steen et al., 2020). (D) Venn diagram illustrating the overlap of differentially expressed genes in MCF10A cells treated with DDR1 and RUNX inhibitors, highlighting a core set of 316 genes impacted by both treatments treated with Col-1 and DDR1 inhibitor (DDR1i) or Col-1 and RUNX inhibitor (RUNX1i), compared to control. (E) Heatmap of the overlapping differentially expressed genes from MCF10A cells, as compared to the control. (F) Unsupervised clustering analysis showing that DDR1i and AI-10-104 treated cells cluster closely together, distinct from control cells, suggesting similar transcriptional responses to both inhibitors. Heatmap of the overlapping differentially expressed genes, as compared to the control. Hierarchically clustered heatmap visualization of the associations between *DDR1-RUNX1* overlapping gene set (input) and enriched transcription factors, utilizing data from the ChEA database (Lachmann et al., 2010). ^∗^ represents transcription factor hits of interest. Statistical significance was determined through multiple t tests, with significance levels indicated as follows ∗*p* < 0.05, ∗∗*p* < 0.01, ∗∗∗*p* < 0.001, ∗∗∗∗*p* < 0.0001.

Given the strong phenotypic similarities observed following DDR1 and RUNX1 inhibition, we sought to investigate whether these effects could be attributed to a common transcriptional network. To test this, we first performed bulk RNA-seq analysis on primary breast organoids, from 3 different donors, treated with DDR1 or RUNX inhibitors. A total of 353 genes were differentially expressed upon DDR1 inhibition and 182 following RUNX inhibition. (Figure 5A).

Unsupervised hierarchical clustering of primary sample-derived organoids showed consistent clustering of treatment groups away from control samples, highlighting similar responses of each patient sample to each inhibitor, and showed the range of effects on *RUNX1* target genes with each treatment (Figure 5B). Among the differentially expressed genes (DEGs), approximately 25% represented direct *RUNX1* transcriptional targets, suggesting a partial but limited overlap. Nineteen of these DEGs from both treatment groups overlapped, indicating a common core of gene targets (Figure 5; Table S3). Using the ChEA dataset (Lachmann et al., 2010) on Harmonize, we found that 94 of the 353 DDR1i-associated genes (∼26%) and 47 of the 182 RUNXi-associated genes (25%) were direct targets of *RUNX1* (Figure S5A).

Gene set enrichment analysis (GSEA) of DEGs revealed loss of *KRAS* and apical signaling as well as expression of *MYC* and *MTOR* targets shared following DDR1i or *RUNX* inhibition (Table S4). GSEA of MicroRNAs (miRNAs) revealed that the differentially expressed gene sets from both DDR1i and RUNXi treatment groups overlapped with genes regulated by miR-93, significantly with miR-93-5p (*p* = 9.27e−3 for DDR1i; *p* = 9.86e−6 for RUNXi), and trending toward significance for miR-93-3p (*p* = 0.0566 for DDR1i; *p* = 0.086 for RUNXi) (Figures S5B and S5C). miR-93 expression is known to be repressed by *RUNX1* and other members of the RUNX TF family and has been implicated in migration and proliferation of normal and cancerous cells (Bao et al., 2020). The large overlap of miR-93-5p target genes in the leading edge for both DDR1i and RUNXi gene sets suggests additional ways *RUNX1* and *DDR1* could share regulatory mechanisms (Figure S5B).

We also applied CIBERSORTx-cell lineage-deconvolution to infer population-level changes (Steen et al., 2020). Both DDR1i and RUNXi treatments significantly reduced the proportions of luminal cell populations (Figure 5C), consistent with the morphological and FACS data (Figures 2F and 4D).

The limited overlap in core transcriptional regulators observed in primary organoids likely reflects patient heterogeneity and long-term adaptations (21 days post-seeding) of primary organoids. Given this, we next examined the transcriptional relationship between *DDR1* and *RUNX1* more directly, using the normal human breast MCF10A cell line exposed to either collagen alone or collagen in combination with DDR1 or RUNX inhibitors. Under these conditions, DDR1 and RUNX inhibition induced highly overlapping transcriptional responses, identifying 316 shared DEGs (representing over half of all DDR1-responsive genes). Over half the genes (316 out of 576) that exhibited differential expression upon DDR1 inhibition overlapped with those affected by RUNX inhibition (Figure 5D). Moreover, unsupervised clustering analysis of these 316 DEGs indicated that cells treated with DDR1i, and cells treated with AI-10-104 closely clustered together, contrasting with the control cells (Figure 5E). Notably, GSEA of the genes within this shared set reveals that they are implicated in known *RUNX1* processes such as tissue development, EMT (Ariffin, 2022), estrogen response (Stender et al., 2010), *MYC* signaling (Choi et al., 2017), as well as numerous other functions (Table S5). This short-term design captured immediate transcriptional responses without confounding compensatory mechanisms, thus revealing a robust common transcriptional network.

We next analyzed the overlapping *RUNX1/DDR1* target gene set to gain deeper insights into how they are regulated at the transcriptional level. (Figures 5F and S5D). We identified several TFs including *SOX2*, *NANOG*, and *OCT4* that regulate the same *RUNX1/DDR1* target genes. These TFs have known roles in the regulating pluripotent stem cell functions as well as cooperating in MCF10A cells with driving stem-like phenotypes (Figures 5F and S5) (Qu et al., 2015; Sokol et al., 2015). Other significant TFs identified as regulating the *DDR1/RUNX1* target genes include *TP63*, *STAT3*, *MYC*, *GATA1*, *TP53*, *E2F1*, and *AR* (Figure 5F). Notably, these TFs regulate stemness and differentiation programs in the breast, but also in other stratified epithelial tissues (Portal et al., 2022; Wu et al., 2003), as well as the hematopoietic system (Aigner et al., 2019; Choi et al., 2017; Huang et al., 2014; Shimizu and Yamamoto, 2023; Trikha et al., 2011). The contrasting results between chronic inhibition in 3D versus acute disruption in MCF10 cells highlight the complexity of *DDR1-RUNX1* signaling dynamics. Acutely, DDR1/RUNX1 share transcriptional responses, while chronically *DDR1/RUNX1* yields subtler transcriptional changes due to cellular adaptation or lineage-specific survival biases. This context-dependent regulation underscores the importance of temporal dynamics and cellular context in the regulation of genes essential for breast epithelial differentiation.

### *DDR1/RUNX1* axis mutations commonly occur in breast cancer

Breast cancer is fundamentally a disease of misregulated development and differentiation—breast cells no longer properly respond to developmental cues and begin to break free from their lineage-restricted behaviors. Breast cancer progression has consequently been linked to the process of de-differentiation. Thus, factors that control differentiation are often among the most frequently mutated genes in breast cancers. The aforementioned results, which indicate that *DDR1* influences a core set of stem cell and differentiation genes by regulating both the expression and interactions of *RUNX1*, also suggest that *RUNX1* and *DDR1* may be altered in breast cancer. Indeed, previous studies have linked both *RUNX1* and DDR1 to specific breast cancer phenotypes within specific subtypes. Both *RUNX1* and *CBFβ* are in the top 30 genes mutated in breast cancer (Ariffin, 2022; Griffith et al., 2018). Expression of *RUNX1* has been hypothesized to be protective to HR^+^ cancers as *RUNX1* has an antagonistic effect on ERα signaling (Stender et al., 2010). Additionally it has been shown that loss of function *RUNX1* somatic mutations occur at a higher rate in estrogen receptor (ER) positive breast cancers, rather than ER negative breast cancers (Ariffin, 2022; Koboldt et al., 2012), further linking this TF to tumor suppressor activity in hormone receptor (HR) positive breast cancers. It is believed that RUNX1 activity stabilizes the epithelial phenotype of the breast tissue (Hong et al., 2017). This observation has been supported by studies using MCF10A cells, where spontaneous *RUNX1* loss in xenograft models is associated with poorly differentiated tumors (Kadota et al., 2010), and targeted knockdown has been shown to lead to hyperproliferation and atypical morphogenesis of spheroids grown from the cell line (Wang et al., 2011). In non-hormonally regulated breast cancers, *RUNX1* expression has been hypothesized to drive increased proliferation (Fernández et al., 2023). Recent studies of DDR1 have shown that its loss plays a role in the epithelial to mesenchymal transition and that higher levels of *DDR1* expression decreases the invasive capacity of a tumor (Koh et al., 2015).

**Figure 6:**
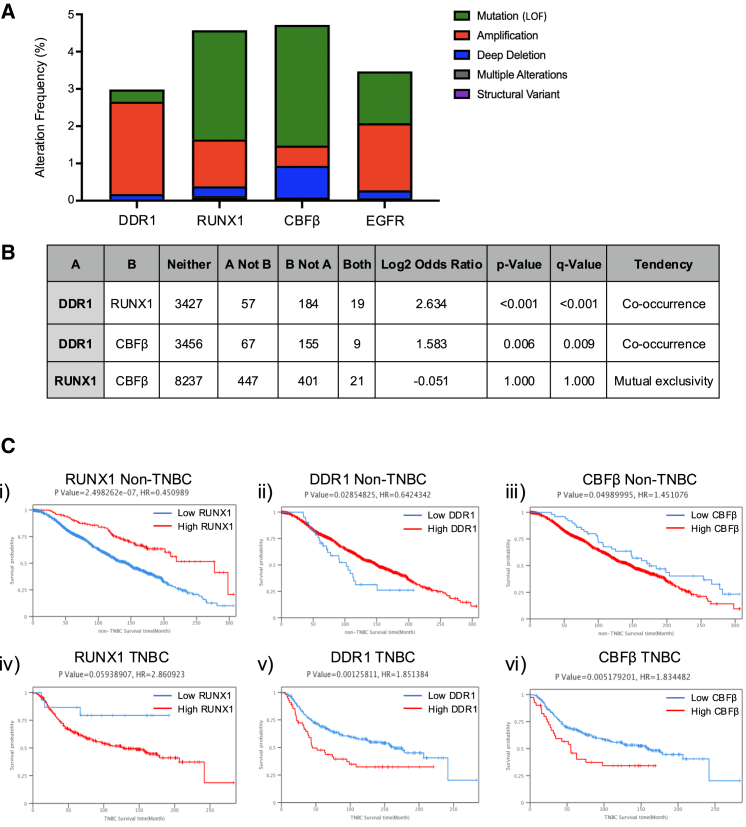
Comprehensive analysis of gene expression and clinical outcomes (A) Genomic alteration frequencies of *RUNX1*, *DDR1*, *CBF*, and *EGFR* in human breast cancer. Mutational frequency from 10,363 breast cancer (BC) samples in 9,776 patients, categorized by mutation type, for genes of interest and well-known regulators in BC, from the cBioPortal database. (B) Mutual exclusivity of *DDR1*, *RUNX1*, and *CBFβ* from the mutational data of 10,363 BC samples in 9,776 patients compiled on the cBioPortal database. (C) Kaplan-Meier OS curve based on high (red) and low (blue) *RUNX1* (i), *DDR1* (ii), or *CBFβ* (iii) in non-triple negative breast cancer (non-TNBC) or TNBC (iv–vi) from METABRIC data on the Breast Cancer Integrative Platform.

To further characterize the effects of the expression and acquired mutations of these proteins in breast cancer subtypes, we utilized the extensive breast cancer database on cBioPortal; in-depth analysis of genetic alteration types by gene demonstrated that *DDR1* alterations in breast cancer were predominantly amplifications (2.48%), while the majority of *RUNX1* and *CBFβ* alterations were mutations (2.93% and 3.24%, respectively) (Figure 6A). These alterations occur at rates such as those seen with epidermal growth factor receptor (*EGFR)* mutations, a commonly occurring mutation, at frequencies between 3% and 5% of all breast tumors (Figure 6A). Further analysis of this database revealed a highly significant tendency for the co-occurrence of mutations in *DDR1* and *RUNX1* as well as in *DDR1* and *CBFβ*, while *RUNX1* and *CBFβ* mutations tend to be mutually exclusive, as expected (Figure 6B). Together, these data suggest that the *DDR1-RUNX1* axis is often perturbed in breast cancer tumors.

Given that *RUNX1* expression has previously been correlated with a positive prognosis in hormone positive non-triple-negative breast cancer (non-TNBC) and negative prognosis in triple-negative breast cancer (TNBC) (Fernández et al., 2023; Koboldt et al., 2012; Stender et al., 2010), we sought to investigate whether *DDR1* and *CBFβ* expression levels exhibit similar correlations. This notion is based on the hypothesis that *DDR1* and *CBFβ* operate within the same pathway as *RUNX1*, contributing to cellular differentiation and structural stability in breast cancer. To assess the association of *DDR1*, *RUNX1*, and *CBFβ* expression levels in these breast cancer subtypes, we used METABRIC data from the Breast Cancer Integrative Platform (BCIP) to produce Kaplan-Meier curves stratifying the association between their expression and survival by breast cancer subtype, with non-TNBC representing luminal/HR^+^ cancers and TNBC representing basal-like cancers. The results showed that similar to the effects of *RUNX1* expression in luminal cancers, elevated expression of DDR1 associated with increased survival in non-triple negative breast cancers (ER^+^/PR^+^/HER2^+^) (Figures 6Ci and 6Cii). In contrast, low expression of *DDR1* was associated with a survival in TNBC, which is similar to what is seen with *RUNX1* expression levels in this subtype (Figures 6Civ and 6Cv). Interestingly, in both cases, low *CBFβ* seemed to be associated with a slight increased survival (Figures 6Ciii–6Cvi). These findings underscore a connection between the expression patterns of *DDR1* and *RUNX1* across different cancer types and their involvement in stem cell states and lineage differentiation. Further work is needed to determine the relevant clinical implications of these proteins for potential therapeutic treatments across different breast cancer subtypes.

## Discussion

The data presented here establish a link between DDR1 activation and the transcriptional activity of *RUNX1* during the development and maturation of breast tissue. Our findings build on previous research about these proteins (Rauner et al., 2021; Sokol et al., 2015), highlighting their critical roles in the early phases of breast organogenesis, specifically during induction and patterning. The failure to activate DDR1 and RUNX1 during these stages hinders cellular lineage proliferation and the formation of alveolar structures. Additionally, our observations show that DDR1 inhibition diminishes protein interaction between RUNX1 and CBFβ. Given CBFβ’s established role in enhancing the affinity between RUNX proteins and DNA (Malik et al., 2019) and the predominant expression of RUNX1 in breast tissue (Mercado-Matos et al., 2017), this disruption significantly impacts the RUNX1-driven transcriptome. Notably, 55% of the genes differentially expressed following DDR1 inhibition in MCF10A cells show changes akin to those observed when RUNX1-CBFβ binding is inhibited. A substantial proportion (54%) of these DEGs are recognized as direct RUNX1 targets, with the remainder potentially influenced by TFs regulated by RUNX1. While we have established a clear connection between DDR1 and RUNX1 modulation through both expression and activity upon inhibition, determining whether this modulation results from a direct or indirect interaction between the two proteins requires further investigation. Future studies are needed to elucidate how the RUNX1-CBFβ complex is regulated downstream of DDR1 activation.

Although further investigation is needed, our data provide valuable insights into the functional roles of RUNX1 in breast tissue, which appear to vary according to the developmental phase and the differentiation state of the cells. During induction, inhibiting the DDR1-RUNX1 axis impedes differentiation, preventing bipotent progenitors and stem cells from progressing to lineage-committed progenitors. In the patterning phase, disruption of signaling through the DDR1-RUNX1 axis obstructs normal physiological morphogenesis. Within the cell, DDR1 inhibition leads to a loss of *RUNX1* mRNA expression in progenitor and basal cell types, resulting in a significant reduction of both RUNX1 and its associated binding partner CBFβ proteins. Previous studies using transplant and organoid models have shown that progenitor and basal cells, which are in direct contact with the extracellular matrix, are the first to develop (Arendt et al., 2010; 2014; Russo and Russo, 2004). Additionally, the effects of RUNX1 and DDR1 inhibition are particularly pronounced in these early cell types when treatment is applied during the initial stages of breast development, highlighting the critical influence of these proteins on stem cells and early progenitors.

The range of functions associated with the identified TFs highlights the dual role that RUNX1 plays downstream of DDR1, involving both the suppression of stem-related genes and the activation of epithelial differentiation genes. A notable observation from our analysis is that nearly 70% of the overlapping gene set (219 out of 316 genes) exhibits upregulation in response to the loss of either *RUNX1* or *DDR1*. This suggests that RUNX1 primarily acts as an inhibitory factor in breast tissue, restraining the transcription of genes that promote a stem-like state, while facilitating the activation of genes necessary for epithelial differentiation. This activity underscores RUNX1’s critical function in maintaining cellular identity and preventing aberrant cell states in breast development.

Recently, RUNX1 and CBFβ have all garnered attention as potential clinical targets in breast cancer (Ariffin, 2022; Han et al., 2022; Malik et al., 2019). Current evidence indicates that *RUNX1* and *CBFβ* are among the 30 most frequently mutated genes in breast cancer (Ariffin, 2022; Griffith et al., 2018; Koboldt et al., 2012), with a prevalence of approximately 4%–5% in breast cancer cases. However, *CBFβ* mutations may be under reported, as deletion of its chromosomal loci at 16q22 represents one of the most frequent and earliest genomic alterations observed in breast cancer, affecting roughly 50% of all cases (Cleton-Jansen et al., 2000). Similarly, *DDR1* mutations in breast cancer, estimated to occur in of 2%–4% of cases, may also be underreported as many genomic studies on breast cancer have not explicitly explored *DDR1*.

Human tumor sequencing data reveals a substantial co-occurrence of mutations between *DDR1* and either *RUNX1* or *CBFβ*, whereas such co-occurrence is not necessarily observed between *RUNX1* and *CBFβ*. These data are in line with our findings, as mutations to *DDR1* and either *RUNX1* or *CBFβ* would have two distinct effects, while loss of both *RUNX1* and *CBFβ* would be largely redundant. In these cases, concurrent loss mutations of *DDR1* and a member of the RUNX1-CBFβ complex could allow for survival advantages by manipulating their role in the balance of stem and differentiated states, or by promoting their normal function of proliferation. Further examination of this co-occurring mutation across all cancer studies on cBioPortal showed that this mutational relationship between DDR1 and either RUNX1 or CBFβ remained strongly significant, indicating that this axis is worth investigating outside of breast cancer. Given the development of potent and selective inhibitors targeting both RUNX1 and DDR1 for potential clinical therapies against various localized tumors, research aimed at deepening our understanding of the role of the DDR1/RUNX1 axis in oncogenesis and normal development is critical.

### Conclusions

Our investigation employing a cutting-edge breast organoid model generated in a 3D hydrogel has shed light on a previously unrecognized yet crucial player within the DDR1 signaling pathway—RUNX1. The activation of DDR1, triggered by the binding of its ligand, collagen, controls the regulation of RUNX1 through *RUNX1* mRNA and protein expression as well as the association between RUNX1 with CBFβ, and thus their downstream transcriptional processes. This newly uncovered DDR1-RUNX1 axis operates as a potent stem cell TF signaling node, orchestrating differentiation, and significantly impacting the morphological characteristics of breast epithelial structures. The clinical implications of these proteins’ expression in cancer are breast cancer subtype specific, warranting further scrutiny to harness the full potential of inhibitor-based therapeutic interventions.

## Methods

### Ethics statement

Primary tissues that normally would have been discarded as medical waste post-surgery were obtained in compliance with all relevant laws, using protocols approved by the institutional review board at Maine Medical Center and Tufts Medical Center. All tissues were anonymized before transfer to prevent tracing back to specific patients; for this reason, this research was provided exemption status by the Committee on the Use of Humans as Experimental Subjects at the Massachusetts Institute of Technology, and at Tufts University Health Sciences (IRB no. 13521). All enrolled patients in this study signed an informed consent form agreeing to participate in this study and for publication of the results.

### 2D cell culture of MCF10 cells

MCF10A (ATCC CRL-10317) and MCF10F (ATCC CRL) cells were cultured in DMEM/F12 (Corning) supplemented with 10 μg/mL insulin (Sigma), 20 ng/mL hEGF (E9644, Sigma Aldrich), 500 ng/mL hydrocortisone (Sigma), 100 ng/mL cholera toxin (Sigma-Aldrich), 5% horse serum (Gibco), and 1× antibiotic/antimitotic (Corning).

### Primary sample preparation

Primary tissue samples from reduction mammoplasties of healthy women were dissociated as previously described (Rauner et al., 2021) Epithelial clusters were dissociated to single cells using 0.25% trypsin-EDTA (Gibco) and filtered through a 40-mm mesh filter after fibroblast removal.

### Collagen stimulation assay

MCF10A and MCF10F cells were seeded at densities of 1e6 cells in 10-cm plates or 2.5e6 cells in 15-cm plates. Cells were treated with vehicle control (DMSO) or with 2 μM DDR1 inhibitor DDR1-in-1 (Tocris, 5077) or RUNX1 inhibitors AI-10-104 (Aobious, AOB17076) at a concentration of 5 μM, RO5-3335 (MedChemExpress, HY-108470) at a concentration of 1 μM, or AI-10-49 (Aobious, AOB0174) at a concentration of 20 nM. After 24 h, a solution of media, collagen (0.05 mg/mL), and 0.1 N NaOH for polymerization, and inhibitor or vehicle was placed on cells during media changes. Twenty-four hours later (48 h of total inhibitor treatment), collagen was removed, cells were lifted with 0.25% trypsin-EDTA, and prepared for downstream assays.

### 3D hydrogel model culture

Single cell primary tissue samples and MCF10A cells were mixed in a suspension of 1.7 mg/mL rat tail collagen I (Corning), 40 μg/mL laminin (Thermo Fisher Scientific), 20 μg/mL fibronectin (Gibco), and 10 μg/mL hyaluronic acid (Sigma), adjusted to pH 7.3 with 0.1 N NaOH. Hydrogels were plated in a four-chamber slide (Falcon) as a mold, polymerized for 1 h at 37°C, and overlaid with MEGM medium (Lonza, CC-3150) supplemented with 1× antibiotic/antimitotic, 1× Glutamax (Gibco). Structures were dissociated with collagenase and trypsin-EDTA, depending on downstream assays.

### Inhibitor time course

Single cell primary tissue samples were seeded into gels in three conditions: control, chronic DDR1 inhibitor or RUNX inhibitor treatment starting day 0 (induction), and chronic DDR1 or RUNX inhibition starting day 7 (patterning). For g-secretase inhibitor DAPT (Sigma, D5942) comparison to DDR1i, done at a concentration of 5 μM, samples developed quickly so they were treated on day 0 for induction and 4 for patterning. Samples were matured for up to 28 days and scored blinded for structure types.

### RNA isolation and quantitative RT-PCR

Cells were pelleted, and RNA was isolated using the RNAeasy kit (QIAGEN). cDNA was produced with the iScript cDNA kit (Bio-Rad), and RT-qPCR was performed with Sybr green (Bio-Rad). Primers for target genes are provided in supplemental information.

### scRNA-seq

Previously published scRNA-seq data from organoids (Rauner et al., 2021) was analyzed using Seurat v3 for data integration, normalization, and feature selection.

### Western blot

Cells grown in 2D or isolated from 3D hydrogels were pelleted by centrifugation and were fractionated using into nuclear and cytoplasmic extracts as previously described (REF). Nuclear fractions were separated via NuPAGE gel (Invitrogen), transferred to PDVF (Bio-Rad) and incubated with primary antibody overnight at 4°C and with secondary antibody for 1 h at room temperature. Immunoblot membranes were developed using a chemiluminescent substrate (Thermo Fisher Scientific) and imaged with the Chemidoc XRS+ with Image Lab 6.0.1 software (Bio-Rad, Hercules, CA). ImageJ2 (version 2.8.0/1.53t) was used to densitometry quantifications. Primary antibodies used were: RUNX1 (4336, Cell Signaling Technology, Clone D33G6, 1:1,000), CBFβ (A303-549A, Bethyl Laboratories, 1:1,000), HDAC1 (5356, Cell Signaling Technology, Clone 10E2, 1:1,000), H3 (9715, Cell Signaling Technology, 1:1,000). Secondary antibodies used were goat anti-rabbit (7074, Cell Signaling Technology, 1:1,000) and goat anti-mouse (7076, Cell Signaling Technology, 1:1,000).

### Co-immunoprecipitation

MCF10A cells from 2D collagen stimulation assays were pelleted by centrifugation and lysed using 1× RIPA buffer containing both 1× protease inhibitor cocktail and 1× phosphatase inhibitor lysate was precleared with protein-A magnetic beads (73778, Cell Signaling Technology). Cells were then incubated in immunoprecipitative antibody overnight at 4°C. Antibodies and attached proteins were conjugated to the magnetic beads at room temperature for 40 min. Samples were separated via SDS-PAGE gel and transferred to PDVF to be blocked in 5% BSA. Blots were then incubated with primary antibody overnight at 4°C and with secondary antibody for 1 h at room temperature. Immunoprecipitative antibody was RUNX1 (HPA004176, SIGMA, 5 μg/mg lysate). Primary antibodies were RUNX1 Ms (sc-365644, Santa Cruz Biotechnology, A-2, 1:1000), CBFβ Ms (67885-1, Proteintech, 1D7F2, 1:1000), and secondary antibody was goat anti-mouse (7076, Cell Signaling Technology, 1:1000).

### Microscopy

Immunofluorescence images captured using Nikon AXR (Nikon Microscopy). Brightfield images captured using Nikon Eclipse Ti-U (Nikon Microscopy), using SPOT 5.6 software.

### Immunofluorescence

Cells and hydrogels were fixed with 4% paraformaldehyde (Thermo Fisher Scientific), permeated with 0.1% Triton 100×, and incubated at 4°C for 18 h with the following primary antibodies: E-Cad (13–1700, Thermo Fisher Scientific, HECD-1, 1:100), CK-14 (RB-9020, Thermo Fisher Scientific, 1:300), CK14 (Abcam, AB7800), ZEB1 (Santa Cruz Biotechnology, sc-25388, 1:100), CK7 (Cell Signaling Technology, 4465, 1:100), EpCAM (Fisher, BD 347200, 1:10,000), and RUNX1(HPA004176, Sigma, 1:100). Samples were then incubated at 4°C for 18 h with the following secondary antibodies: DAPI (D1306, Life Technologies, 1:1000), AF488 (A11008, Invitrogen, 1:1000), AF555 (A21424, Invitrogen, 1:1000), and Phalloidin-AF647 (A22289, Invitrogen, 1:500).

### Live imaging

Primary single cells, isolated as described previously, were incubated with the cell tracking dye Cytopainter Green (1:500, cat no. ab138891, Abcam) for 30 min and then were washed and seeded at a concentration of 100 cells per 20 μL hydrogel. For gel fabrication, 20 μL hydrogel drops were deposited onto the center wells of a 96-well plate (Corning, no. 3603). Gels were allowed to incubate for one hour at 37°C until fully polymerized. MEGM (80 μL) was then added to each well and gels were gently lifted off the well surface with a pipette tip. Cultures were immediately placed in a pre-warmed incubator chamber (Okolab Inc.) enclosed over a Nikon Eclipse Ti2-AX confocal microscope (Nikon Microscopy). Images of selected points were collected starting immediately after the addition of media, and every 30–45 min after, in both brightfield and with A488 laser at 4× magnification and 2.5× zoom across nine *z* positions. MEGM (20–40 μL) was added to the culture twice a week to maintain proper growth factors and liquid volume to prevent hydrogels from drying out. Cultures were live imaged for 18–21 days. Analysis and production of videos across locations and time points was performed using NIS-Elements (Nikon) and Premiere Pro (Adobe) software.

### Flow cytometry

Patient sample-derived structures, either control or inhibited during patterning at day 7, were dissociated and pelleted by centrifugation. Samples were washed and stained with the antibodies CD49f-FITC (555736, BD Biosciences, GoH3, 1:20) and EPCAM-PE (347198, BD Biosciences, 1:20). Samples were run on LSRII. FlowJo (version 10.9.0) was used for visualization and quantification.

### RNA-seq

RNA-seq was performed on human breast epithelial organoids and MCF10A cells to identify transcriptional changes upon DDR1 and RUNX inhibition. Organoids derived from primary human breast epithelial cells (3 different donor samples) were seeded as single cells into 3D hydrogels. Treatments with DDR1 inhibitor (DDR1i) or the RUNX inhibitor AI-10-104 began at day 7 of organoid development and continued until day 21. Organoids were allowed to form and grow until day 21, at which point they were extracted from the collagen matrix, lysed, and processed for RNA extraction and bulk RNA-seq. For MCF10A cells, mRNA isolated from cells in a collagen stimulation assay for 48 h. Organoids or MCF10A cells were lysed, and RNA was extracted using the RNeasy Mini Kit (QIAGEN), following the manufacturer’s protocol. RNA integrity and concentration were assessed using a bioanalyzer (Agilent). Libraries were prepared using TruSeq RNA Library Preparation kits (Illumina), and sequencing was performed on an Illumina NovaSeq platform, generating 150-bp paired-end reads. Data quality was checked using FastQC, followed by alignment to the human genome using STAR.

### MCF10A RNA-seq analysis

Read alignment to the human genome was performed using STAR with the CRCh37/hg19 assembly. Library normalization as well as differential expression testing was performed using R with DESeq2 (3.17). Sample SSR107 was removed as it was deemed outlier by PCA. Differential gene analysis was conducted with significance determined by log fold change greater than or less than 0 with a *p* value of less than 0.05. Gene ontology analysis of the 316 overlapping gene set was performed using the ChEA dataset (Lachmann et al., 2010) visualized with Harmonize (version 3.0) and with the TRRUST (Han et al., 2015) dataset from ENRICHR to gain deeper insights into the potential cellular processes governed by the shared 316 DDR1/RUNX target genes. Data were also input into GSEA to determine hallmark gene sets that may be affected. Heat maps and Venn diagrams were created in R using Pheatmap and ggVennDiagram, respectively.

### Organoid RNA-seq analysis

Read alignment to the human genome was performed using STAR with the GRCh38 p14 assembly. Genes were retained for downstream analysis only if at least two out of nine samples exhibited non-zero counts. Differential gene expression analysis was performed using DESeq2 to compare paired patient samples, with significance determined by log fold change greater than or less than 0, and by a Benjamini-Hochberg adjusted *p* value of less than 0.05. TF targets’ analysis of the DEGs was performed using the ChEA dataset (Lachmann et al., 2010) on Harmonize (version 3.0) and visualized with R. GSEA was conducted using the C3:miR gene set collection from the Molecular Signatures Database. RNA-seq deconvolution analysis was conducted using CIBERSORTx (Steen et al., 2020) with a reference single cell gene set created from the epithelial populations extracted from single-cell sequencing of healthy breast tissue (Gray et al., 2022) and visualized with R.

### Mutational analysis

RUNX1, CBFβ, and DDR1 were probed for breast cancer alteration frequency on cBioPortal using primary tumor BC data from 20 studies broken down by cancer type. Mutational status across breast cancer subtype by PAM50 and exploration into mutational mutual exclusivity were also conducted using these studies on cBioPortal. Kaplan Meier survival curves were produced using METABRIC data the BCIP, plotting overall survival based upon transcriptome analysis of either a triple negative status, or non-triple negative (i.e., the expression of at least one receptor ER/Progesterone Receptor/HER2).

### Statistics

All statistics were performed using GraphPad Prism 8–10. Student’s t tests (two sided) were performed as a determinant of significance unless otherwise stated. Data are expressed as mean ± SD. Significance levels are indicated as follows: ^∗^*p* < 0.05, ^∗∗^*p* < 0.01, ^∗∗∗^*p* < 0.001, and ^∗∗∗∗^*p* < 0.0001.

## Resource availability

### Lead contact

Information requests can be directed to the lead contact, Charlotte Kuperwasser (charlotte.kuperwasser@tufts.edu).

### Materials availability

Materials used in this study are available from the lead contact, Charlotte Kuperwasse, upon request.

### Data and code availability

This paper analyzes two existing scRNA-seq datasets, publicly available through the following GEO accession numbers: GSE180878 and GSE162296. Original scRNA-seq data for this paper was deposited to GEO, and is available through the following accession numbers: GSE298818 and GSE272979. Any additional information required to reanalyze the data reported in this paper is available from the lead contact upon request.

## Acknowledgments

We gratefully acknowledge Albert Tai, Irena Grinvald, and Michael Berne at the Tufts Genomics core for high-throughput sequencing services; Stephen Kwok and Allen Parmelee at the Tufts Laser Cytometry Core Facility for flow cytometry support; and Karla Murga, Daniela Requena, and Megan Maloney at Tufts Biomedical Repository for tissue support. This research was supported by the following: 10.13039/100000002NIH/10.13039/100000057NIGMS (7R01GM124491 to P.B.G. and C.K.), 10.13039/100000002NIH/10.13039/100000057NIGMS (T32GM150533 to R.W.), 10.13039/100001006Breast Cancer Research Foundation (to C.K.), and Find The Cause (FTC) Breast Cancer Foundation (to C.K. and S.M.).

## Author contributions

C.T., G.R., N.T., M.P., D.E.C.F., Y.M., P.B.G., and C.K. conceived the project and designed experiments. C.T. performed experiments. C.T. and N.T. performed sequencing analysis. R.W. and S.M. performed RNA-seq data analysis and interpretation. C.T. and C.K. wrote the manuscript.

## Declaration of interests

G.R. consults for Turtle Tree Inc. C.K. is co-founder and consultant of Naveris Inc. P.B.G. is co-founder, Chief Science and Technology Officer, and Executive Chairman of Naveris Inc.

## Supplemental information

Document S1. Figures S1–S5 and Tables S1–S5

Document S2. Article plus supplemental information
