# Supplementary material for: DDR1 regulates RUNX1-CBFβ to control breast stem cell differentiation
Source: Stem Cell Reports. 2025 Jul 3;20(8):102576. doi: 10.1016/j.stemcr.2025.102576 (PMC12365826; doi:10.1016/j.stemcr.2025.102576)
Supplement: Document S1. Figures S1–S5 and Tables S1–S5 [file mmc1.pdf]

Stem Cell Reports, Volume *20*

Supplemental Information

DDR1 regulates RUNX1-CBFβ to control breast stem cell differentiation

Colin Trepicchio, Gat Rauner, Nicole Traugh, Ruohong Wang, Meadow Parrish, Daniel E. C. Fein, Youssof Mal, Piyush B. Gupta, Stefano Monti, and Charlotte Kuperwasser

Supplemental Materials

Quantitative RT-PCR Primers: ABCD4: Forward: TGCGTACTACACCCTCAACG; ReverseTGAACGGGGAGATGATGAG. BCAR3: Forward: TCAGGGATCCACATCTTCTG; ReverseCCAGCTCCTTCTTCAGTTTCTC, ZEB1: Forward: TAAGAACTGCTGGGAGGATGAC; ReverseTCTGCATCTGACTCGCATTC

RNAseq: raw data was loaded and integrated into one Seurat object using the merge function. Filtering removed cells with < 200 or >2500 genes and mitochondrial content greater than 7.5%. Genes detected in less than 3 cells were dropped from analysis. The data was normalized by multiplying transcripts by a factor of 10,000 followed by log-transforming the data. Variable features used for analysis were identified by using the FindVariableFeatures function, with a low cutoff of 0.0125 and a high cutoff of 5 for dispersion and a low cutoff of 0.1 and a high cutoff of 0.8 for average expression. The data was integrated by the FindIntegrationAnchors and IntegrateData functions, which identify the anchors to integrate the two datasets, and then integrates them together. Cells were then clustered using K-nearest neighbor (KNN) graphs and the Louvain algorithm using the first 10 dimensions from principal component analysis. Clustered cells were visualized by tSNE embedding using the default settings in Seurat. Clusters were called using the FindClusters function with a resolution of 1. To identify differentially expressed genes between cell clusters, we utilized the FindAllMarkers function to identify features detected in >10% of a cell cluster compared to all other cells. Pathway analysis to identify enriched biological pathways associated with differentially expressed genes was done using established databases, such as PanglaoDB. The top 15 differentially expressed markers were used to determine gene expression location.

Supplemental Figure 1: Effects of DDR1i on single-cell-derived breast organoid development

A) Quantification of the total number of organoids that formed following DDR1i treatment during induction. Data presented as Mean ± SD (n= 4 gels/ 2 primary patient samples). B) Representative immunofluorescent panels showing the expression of EMT transcription factor ZEB1in structures treated with DDR1i during induction. ZEB1 (Green), Phalloidin (Red), DAPI (Blue). Scale bar = 20 μm. C) Quantification of the total number of organoids that formed following DDR1i treatment during

Patterning. Data presented as Mean ± SD (n= 4 gels / 3 primary patient samples). Statistical significance was determined through multiple t-tests, with significance levels indicated as follows: *p-value < 0.05, **p-value < 0.01, ***p-value < 0.001, ****p-value < 0.0001.

Supplemental Figure 2: RUNX1 expression across development

Representative immunofluorescent images showing RUNX1 expression in the nuclei of most cells during early patterning, morphogenesis, and mature stages of development. RUNX1 (Green), Phalloidin (Red), DAPI (Blue).

Supplemental Figure 3: Differential expression analysis in response to DDR1 inhibition.

A) scRNA-seq clustering of epithelial cells from primary tissue organoids. B) Violin plots showing the distribution of DDR1 expression across epithelial breast cell types. C-J) Violin plots showing the distribution of expression in RUNX1 target genes: ANP32B, PLEC, CEBPD, ID1, STAT3, and the expression of related transcription factors RUNX2, RUNX3 and CBFβ across epithelial clusters in primary tissue organoids grown for 14 days under control DDR1i or DDR1r conditions. K) Quantification of RUNX1 target gene expression from three different patient samples (i-iii). Data expressed as Mean ± SD. L**)** Quantification of RUNX1 target gene expression from MCF10F cells in a 2D collagen stimulation assay. Data expressed as Mean ± SD. Statistical significance was determined through multiple t-tests, with significance levels indicated as follows: *p-value < 0.05, **p-value < 0.01, ***p-value < 0.001, ****p-value < 0.0001.

Supplemental Figure 4: Effects of RUNXi on breast organoid development

A) Western blot showing co-immunoprecipitation of CBFβ bound to the pulled down RUNX1 when exposed to DDR1i and RUNX inhibitors AI-10-104, AI-10-47, and RO5-3335. B) Quantification of the total number of organoids that formed following AI-10-104 treatment during induction. Data presented as Mean ± SD (n= 4 gels/ 2 primary patient samples). C) Representative brightfield images depicting

primary patient samples cultured in a 3D environment, comparing control conditions with DDR1 and RUNX inhibitor-treated conditions initiated during patterning. Scale bar = 200 µm. D) Quantification of the total number of organoids that formed following AI-10-104 treatment during Patterning. Data presented as Mean ± SD (n= 4 gels/ 3 primary patient samples). E) Normalized quantification of different structure types; (i) Alveolar, (ii) Ductal, (iii) Compound, formed when exposed to DDR1 or NOTCH1 inhibitors (gsi) beginning during induction or patterning. Data presented as Mean ± SD (n= 4 gels). F) Normalized quantification of different structure types; (i) Alveolar, (ii) Ductal, (iii) Compound, formed when exposed to DDR1-in-1 (DDR1i) or DAPT (NOTCH1i) treatments either beginning during induction or patterning. Data presented as Mean ± SD (n= 8 gels/ 2 primary patient samples). Statistical significance was determined through multiple t-tests, with significance levels indicated as follows: *p-value < 0.05, **p-value < 0.01, ***p-value < 0.001, ****p-value < 0.0001.

Supplemental Figure 5: Transcription factor insights into core DDR1-RUNX1 gene set

A) Pie charts comparing the number of differentially expressed genes within three patient samples, compared to control, that are known RUNX1 transcriptional target genes with either DDR1i or RUNXi treatments. B) Mountain plots depicting the distribution of miR-93 5p target genes within the ranked list of genes differentially expressed following either DDR1 or RUNX1 inhibition. Venn Diagram depicts the overlap of genes found in the leading edge. C) Mountain plots depicting the distribution of miR-93 3p target genes within the ranked list of genes differentially expressed following either DDR1 or RUNX1 inhibition. Venn Diagram depicts the overlap of genes found in the leading edge. D**)** Hierarchically clustered heatmap presenting the association between various transcription factor hits and genes differentially expressed from our overlapping gene set (input), utilizing data from the TRRUST database. Red squares indicate that the associated transcription factor is known to regulate the gene in this dataset. Generated with ENRICHR.

Supplemental Table 1: List of samples

List of samples used for each experiment and notes on sample type, age, body mass index (BMI), and other factors that may affect the growth of the primary sample.

Supplemental Table 2: scRNA-Seq lineage groupings

List of top differentially expressed genes used to create groupings for scRNA-Seq Data. Basal classifications were collapsed into one group for plotting on graph.

Supplemental Table 3: Differential gene expression of primary samples

A). List of co-occurring differentially expressed genes that move in the same direction with DDR1i and RUNXi treatments, in all three patient samples.

Supplemental Table 4: GSEA analysis of DDR1-RUNX1 transcriptome

A). Gene set enrichment analysis (GSEA) interrogating core DDR1-RUNX1 gene set with the top 20 Hallmark Gene sets from Molecular Signatures Database (MSigDB)

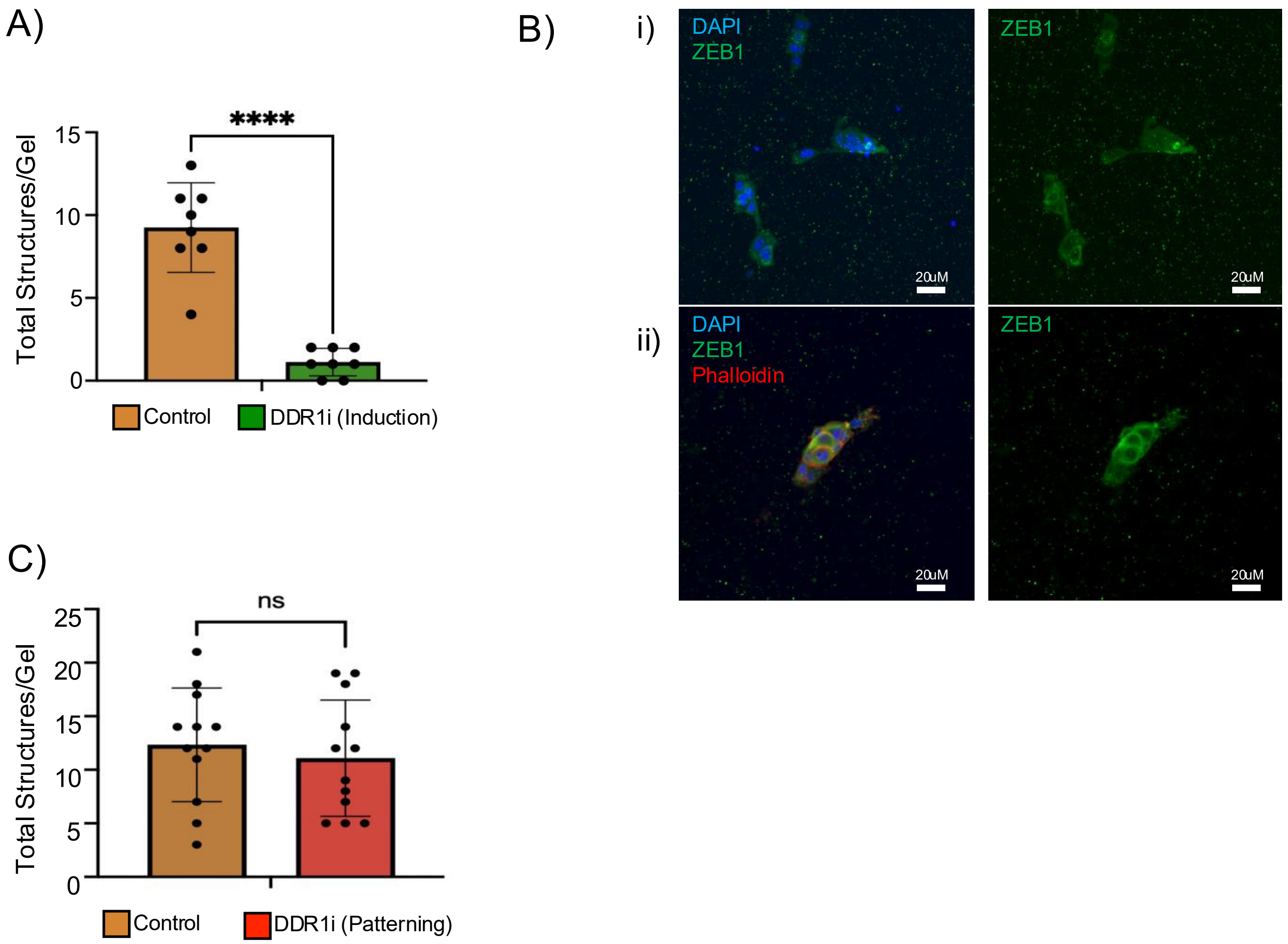

Supplemental Figure 1

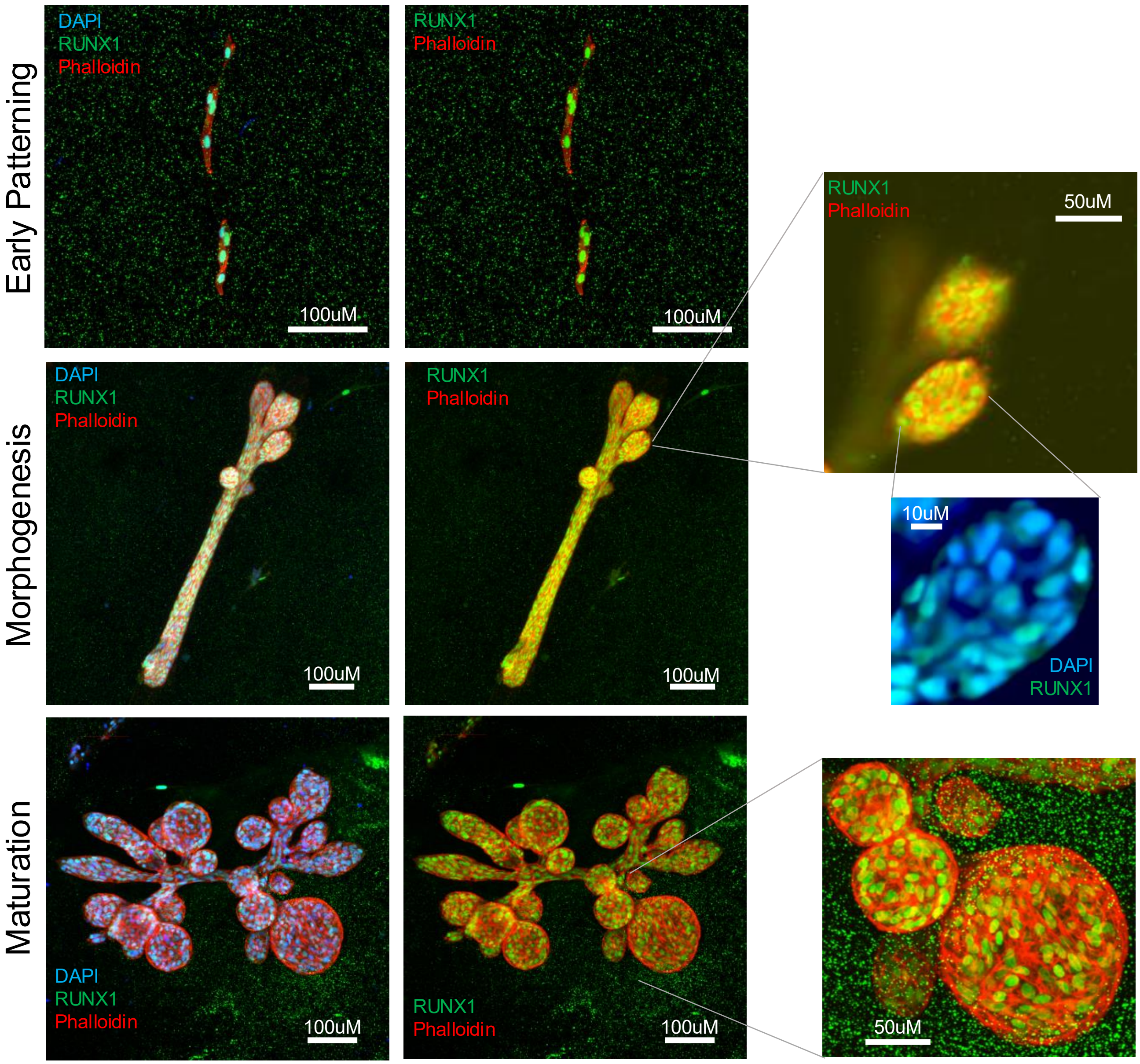

Supplemental Figure 2

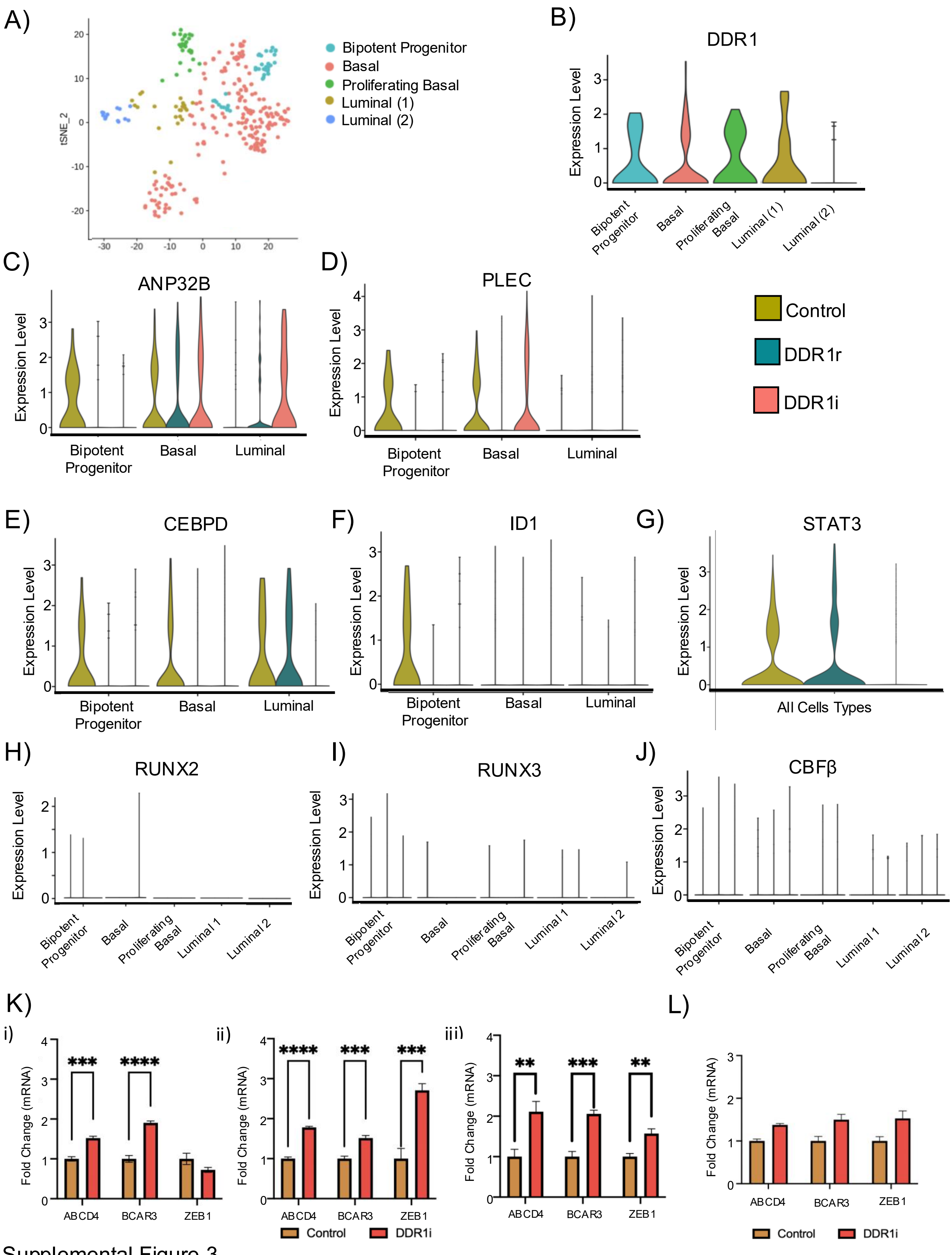

Supplemental Figure 3

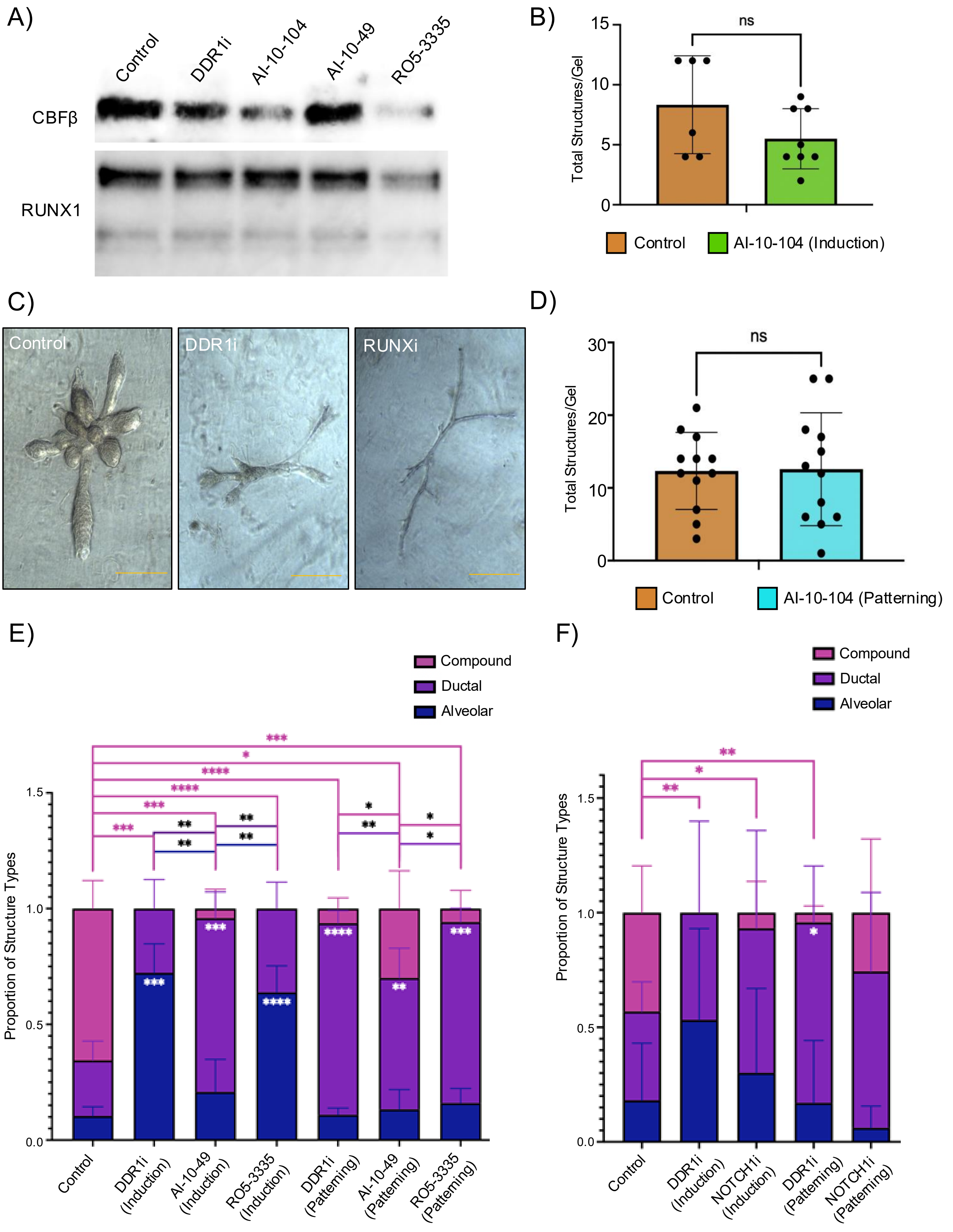

Supplemental Figure 4

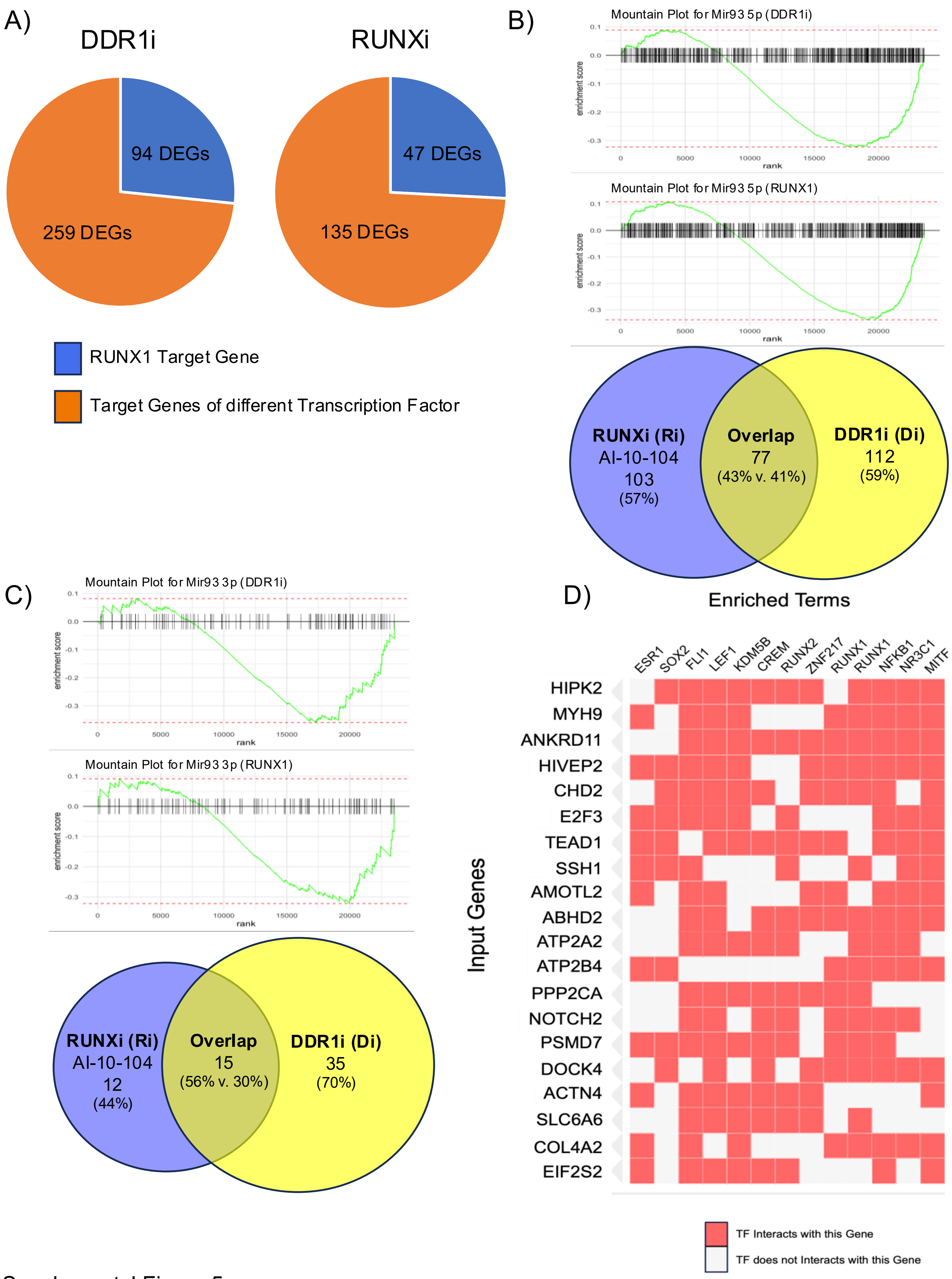

Supplemental Figure 5

A)

Sample	Sample Type	Experiments Used in	Age	Other
**683**	Reduction Mammoplasty	Structure Quantification, Immunofluorescence, Live Imaging, FACS, Sequencing	49	• Normal/Healthy
**686**	Reduction Mammoplasty	Structure Quantification, Immunofluorescence, Live Imaging, Western Blot, FACS, Sequencing	32	• Normal/Healthy
**687**	Reduction Mammoplasty	Structure Quantification, Immunofluorescence, Live Imaging, Western Blot, qRT-PCR, FACS	38	• Normal/Healthy
**696**	Reduction Mammoplasty	Structure Quantification, Immunofluorescence, Live Imaging, Western Blot, FACS, Sequencing	56	• BMI of 32.2
**697L**	Double Mastectomy	Structure Quantification, qRT-PCR	19	• Right Breast (697R) had an atypical papilloma<br>• BMI 27.21
**700R**	Reduction Mammoplasty	Structure Quantification, qRT-PCR	51	• BMI of 27.37
**805**	Reduction Mammoplasty	Structure Quantification, Immunofluorescence, Live Imaging	20	• BMI of 28.06

Supplemental Table 1

A)

Gene	Bipotent Progenitor	Basal	Basal	Basal	Basal	Proliferating Basal	Luminal (1)	Luminal (2) *Mature*
**1**	KRT6A	MYLK	RPL3P2	MALAT1	S100A2	ASPM	KRT19	STAC2
**2**	KRT16	SPARC	RPL23	NEAT1	KRT14	MKI67	AZGP1	GLYATL2
**3**	GJB2	A2M	UBE2N	AP000769.1	ITGA6	TOP2A	KRT18	SAA2
**4**	KRT17	SPARCL1	RPL26	XIST	KRT5	NUSAP1	KLK6	SCGB1D2
**5**	KRT6B	SESN3	RPS20	FOSB	LAMB3	TPX2	RARRES1	CCL28
**6**	CCNA1	GJA1	SUMO2	FUS	SFN	BUB1	KRT81	AZGP1
**7**	LGALS7B	DSC3	RPL15	NKTR	LAMA3	DLGAP5	C6orf15	CLDN3
**8**	FAM84A	TAGLN	MYL6	CHD2	LAMC2	CENPF	MGP	RBM47
**9**	PERP	VCAN	RNF181	LDLR	AREG	CENPE	KRT8	SYNM
**10**	SLPI	PTN	CALML3	SREK1	GSTP1	DEPDC1	WFDC2	RCAN1
**11**	DSG3	PLAT	CST3	SRRM2	PRNP	ANLN	KRT7	LTF
**12**	CARD18	DKK3	KRT15	SARAF	KRT6A	CCNB1	KLK7	SERPINA3
**13**	SERPINB5	CLCA2	RALA	PNISR	CALML3	KIF11	CLDN4	PLEKHS1
**14**	DSP	SERPINH1	S100A2	PNN	ODC1	HMMR	CD24	NR4A1
**15**	SH3KBP1	FHDC1	KRT14	CCNL1	CSTA	NCAPG	EPCAM	DEFB1

Supplemental Table 2

A)

Gene	log2FoldChange.DDR1i	padj.DDR1i	log2FoldChange.RUNXi	padj.RUNXi
LINC00342	-2.2277772	0.00730725	-2.5986804	0.00168861
APPAT	-1.8329045	0.0199586	-2.6239684	0.00031695
PPFIA4	-1.7052535	0.02352877	-1.9028483	0.0120125
NPIPA6	-1.3003987	0.02392481	-1.5471814	0.00555912
WASH5P	-1.1739841	0.0386013	-1.4229456	0.00856563
DUOX1	-1.109003	0.00041978	-1.2129564	0.00022171
DDX39B	-1.0835731	0.0229621	-1.2830346	0.00554489
WSB1	-1.0374469	0.01210898	-1.334566	0.00062599
NKTR	-0.9733074	0.03783515	-1.3282731	0.00159329
FAT2	-0.9591466	0.00093764	-1.1903583	4.3221E-05
XYLT1	-0.94433	7.5676E-06	-1.132478	2.0935E-07
PCSK9	0.92483677	0.00482072	-1.3019287	5.2728E-05
NQO1	1.00361635	7.924E-05	0.65748	0.04122437
AKAP12	1.0386018	0.01887441	1.42772283	0.00043382
APOD	1.10369259	0.01997604	1.26522997	0.0071036
GFPT2	1.81209321	0.04938445	2.22884038	0.00967602
HS3ST2	1.8555124	8.4903E-08	1.2165303	0.00433206
TAC1	2.94425925	0.01376926	3.55618317	0.00184531
ESM1	5.56295462	0.04602164	5.99388932	0.02940666

Supplemental Table 3

Gene Set	DDR1i overlap	DDR1i pval	DDR1i fdr	RUNXi overlap	RUNXi pval	RUNXi fdr	Up or Down Regulated
HALLMARK_KRAS_SIGNALING_DN	161	1.4E-12	1.4E-11	161	0.08	0.12	Down
HALLMARK_APICAL_JUNCTION	193	0.0063	0.017	193	0.017	0.03	Down
HALLMARK_APICAL_SURFACE	41	0.056	0.094	41	0.23	0.27	Down
HALLMARK_WNT_BETA_CATENIN_SIGNALING	40	0.18	0.25	40	0.29	0.32	Down
HALLMARK_IL2_STAT5_SIGNALING	188	0.21	0.28	188	0.2	0.24	Down
HALLMARK_INTERFERON_ALPHA_RESPONSE	97	0.28	0.34	97	0.18	0.23	Down
HALLMARK_HEDGEHOG_SIGNALING	36	0.44	0.45	36	0.0035	0.0076	Down
HALLMARK_OXIDATIVE_PHOSPHORYLATION	200	1E-33	5E-32	200	0	0	Up
HALLMARK_MYC_TARGETS_V1	199	1.4E-30	3.6E-29	199	0.00000061	0.0000022	Up
HALLMARK_MTORC1_SIGNALING	199	1.1E-19	1.9E-18	199	0.00017	0.00048	Up
HALLMARK_CHOLESTEROL_HOMEOSTASIS	74	6.7E-10	5.6E-09	74	0.095	0.14	Up
HALLMARK_FATTY_ACID_METABOLISM	151	2.2E-08	0.0000001<br>6	151	0.0000037	0.000012	Up
HALLMARK_DNA_REPAIR	150	0.0000002	0.0000013	150	0.00000011	0.00000047	Up
HALLMARK_ADIPOGENESIS	195	0.00000027	0.0000015	195	0	1.9E-10	Up
HALLMARK_REACTIVE_OXYGEN_SPECIES_PATHWAY	47	0.00000065	0.0000032	47	1.3E-08	0.00000007	Up
HALLMARK_UNFOLDED_PROTEIN_RESPONSE	111	0.00011	0.00038	111	0.13	0.17	Up
HALLMARK_MYC_TARGETS_V2	57	0.00017	0.00056	57	0.26	0.3	Up
HALLMARK_PEROXISOME	98	0.0077	0.019	98	0.022	0.036	Up
HALLMARK_KRAS_SIGNALING_UP	187	0.0098	0.023	187	0.000053	0.00016	Up
HALLMARK_MYOGENESIS	180	0.012	0.028	180	0.13	0.17	Up
HALLMARK_PI3K_AKT_MTOR_SIGNALING	100	0.016	0.033	100	0.0013	0.003	Up
HALLMARK_ANDROGEN_RESPONSE	99	0.033	0.061	99	0.011	0.022	Up
HALLMARK_COMPLEMENT	186	0.089	0.14	186	0.0000042	0.000013	Up
HALLMARK_BILE_ACID_METABOLISM	104	0.13	0.19	104	0.19	0.23	Up
HALLMARK_IL6_JAK_STAT3_SIGNALING	78	0.36	0.4	78	0.017	0.03	Up
HALLMARK_PROTEIN_SECRETION	96	0.43	0.45	96	0.11	0.15	Up

Supplemental Table 4

A)

Hallmark Gene Set	# of Bookmarked Genes	p-value	FDRq-value
**Mitotic Spindle**	21	$3.03\ e^{-18}$	$1.51\ e^{-16}$
**Myc Targets**	18	$1.34\ e^{-14}$	$3.36\ e^{-13}$
**Oxidative Phosphorylation**	14	$3.48\ e^{-10}$	$5.81\ e^{-9}$
**Apical Junction**	11	$3.21\ e^{-7}$	$3.21\ e^{-6}$
**Epithelial Mesenchymal Transition**	11	3.21 e-7	3.21 e-6
**IL2 Stat5 Signaling**	10	$2.5\ e^{-6}$	$2.09\ e^{-5}$
**E2F Targets**	9	$1.93\ e^{-5}$	$1.07\ e^{-4}$
**Estrogen Response Early**	9	$1.93\ e^{-5}$	$1.07\ e^{-4}$
**Estrogen Response Late**	9	$1.93\ e^{-5}$	$1.07\ e^{-4}$
**NOTCH Signaling**	4	$8.75\ e^{-5}$	$4.38\ e^{-4}$
**Myogenesis**	8	$1.27\ e^{-4}$	$5.77\ e^{-4}$
**TGF Beta Signaling**	4	$6.78\ e^{-4}$	$2.31\ e^{-3}$
**UV Response (Down)**	6	$7.17\ e^{-4}$	$2.31\ e^{-3}$
**G2M Checkpoint**	7	$7.4\ e^{-4}$	$2.31\ e^{-3}$
**MTORC1 Signaling**	7	$7.4\ e^{-4}$	$2.31\ e^{-3}$
**TNFA Signaling vis NFkB**	7	$7.4\ e^{-4}$	$2.31\ e^{-3}$
**Complement**	6	$3.77\ e^{-3}$	$1.11\ e^{-2}$
**Myc Targets V2**	3	$9.01\ e^{-3}$	$2.5\ e^{-2}$
**Heme Metabolism**	5	$1.65\ e^{-2}$	$4.13\ e^{-2}$
**P53 Pathway**	5	$1.65\ e^{-2}$	$4.13\ e^{-2}$

Supplemental Table 5
